# Temporal, Spatial, and Temperature Controls on Organic Carbon Mineralization and Methanogenesis in Arctic High-Centered Polygon Soils

**DOI:** 10.3389/fmicb.2020.616518

**Published:** 2021-01-11

**Authors:** Taniya Roy Chowdhury, Erin C. Berns, Ji-Won Moon, Baohua Gu, Liyuan Liang, Stan D. Wullschleger, David E. Graham

**Affiliations:** ^1^Oak Ridge National Laboratory, Biosciences Division, Oak Ridge, TN, United States; ^2^Oak Ridge National Laboratory, Environmental Sciences Division, Oak Ridge, TN, United States; ^3^Oak Ridge National Laboratory, Climate Change Science Institute, Oak Ridge, TN, United States

**Keywords:** anaerobic carbon mineralization, methanogenesis, mcrA, permafrost, Arctic tundra

## Abstract

Warming temperatures in continuous permafrost zones of the Arctic will alter both hydrological and geochemical soil conditions, which are strongly linked with heterotrophic microbial carbon (C) cycling. Heterogeneous permafrost landscapes are often dominated by polygonal features formed by expanding ice wedges: water accumulates in low centered polygons (LCPs), and water drains outward to surrounding troughs in high centered polygons (HCPs). These geospatial differences in hydrology cause gradients in biogeochemistry, soil C storage potential, and thermal properties. Presently, data quantifying carbon dioxide (CO_2_) and methane (CH_4_) release from HCP soils are needed to support modeling and evaluation of warming-induced CO_2_ and CH_4_ fluxes from tundra soils. This study quantifies the distribution of microbial CO_2_ and CH_4_ release in HCPs over a range of temperatures and draws comparisons to previous LCP studies. Arctic tundra soils were initially characterized for geochemical and hydraulic properties. Laboratory incubations at −2, +4, and +8°C were used to quantify temporal trends in CO_2_ and CH_4_ production from homogenized active layer organic and mineral soils in HCP centers and troughs, and methanogen abundance was estimated from *mcrA* gene measurements. Results showed that soil water availability, organic C, and redox conditions influence temporal dynamics and magnitude of gas production from HCP active layer soils during warming. At early incubation times (2–9 days), higher CO_2_ emissions were observed from HCP trough soils than from HCP center soils, but increased CO_2_ production occurred in center soils at later times (>20 days). HCP center soils did not support methanogenesis, but CH_4_-producing trough soils did indicate methanogen presence. Consistent with previous LCP studies, HCP organic soils showed increased CO_2_ and CH_4_ production with elevated water content, but HCP trough mineral soils produced more CH_4_ than LCP mineral soils. HCP mineral soils also released substantial CO_2_ but did not show a strong trend in CO_2_ and CH_4_ release with water content. Knowledge of temporal and spatial variability in microbial C mineralization rates of Arctic soils in response to warming are key to constraining uncertainties in predictive climate models.

## Introduction

**FIGURE 1:**
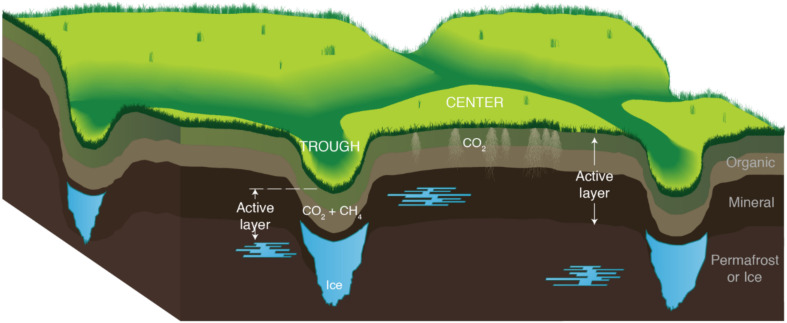
Conceptual illustration of the soil profile in high-center polygon (HCP) troughs and centers. Melting ice wedges (blue) cause the trough areas to subside, promoting drainage from the centers that saturates the troughs. The organic and mineral soil layers comprise the active layer, which thaws annually to the top of the permafrost (horizons noted on the right side of figure). Locations of CO_2_ and CH_4_ production are also indicated. The lateral scale of the polygons is much larger than the exaggerated vertical scale.

Arctic warming will transform tundra ecosystems by increasing soil temperatures and active layer depth, lengthening the annual active layer thaw period, and releasing large quantities of soil organic carbon (SOC) (Shaver et al., 1992; Schuur et al., 2009; Waldrop et al., 2010; Yergeau et al., 2010; Mackelprang et al., 2011; Lipson et al., 2013; Olefeldt et al., 2013). These changes could promote microbial degradation of SOC, increasing the flux of carbon dioxide (CO_2_) and methane (CH_4_) from Arctic soils and accelerating climate warming trends (Tveit et al., 2013; Crowther et al., 2015; Tang et al., 2016; Pries et al., 2017; Jansson and Hofmockel, 2019). Polygonal – or patterned – landscapes, formed by expanding ice wedges, are common in many regions of Arctic tundra and contribute to carbon (C) release during warming. Massive ice wedges form in cracks present in drained, interstitial tundra and give rise to low centered polygons (LCPs), which are typically characterized by water-saturated centers surrounded by elevated rims and outlying troughs. As ice wedges degrade, drier high centered polygons (HCPs) are formed, which drain water outward to surrounding troughs (Figure 1). Utqiagvik (formerly Barrow), Alaska is located in a region dominated by thaw lakes and polygonal tundra. LCP centers, rims, and troughs make up approximately 24% of the tundra (Billings and Peterson, 1980; Hinkel and Nelson, 2003; Walker et al., 2008), whereas HCP centers and troughs comprise 11% (Lara et al., 2015; Liljedahl et al., 2016). To effectively model and evaluate warming-induced CO_2_ and CH_4_ fluxes from tundra soils in regions like Utqiagvik, data quantifying CO_2_ and CH_4_ release from both LCP and HCP soils are needed. Controlled laboratory incubation studies are routinely used to identify the key drivers of gas emission patterns and the role of microbial properties like gene abundance, that in turn can inform model parameterization (Evans and Wallenstein, 2014; Faucherre et al., 2018; Philben et al., 2020a).

The microtopographic variability of polygonal landscapes controls hydrological heterogeneities, which have been shown to correlate with differences in pH, dissolved oxygen (DO), ferrous iron [Fe(II)], nutrients, and microbial activity (Fiedler et al., 2004; Preuss et al., 2013; Herndon et al., 2015, 2020; Lipson et al., 2015; Newman et al., 2015; Taş et al., 2018; Wu et al., 2018). These biogeochemical gradients across LCPs and HCPs may control the nature and magnitude of C flux from Arctic soils. Previous research has shown variability in CO_2_ and CH_4_ release across HCPs and LCPs and their associated microtopographies (trough, rim, and center) (Lipson et al., 2012; Roy Chowdhury et al., 2015; Wainwright et al., 2015; Grant et al., 2017; Taş et al., 2018), and some studies have begun to develop relationships between hydrological and biogeochemical controls on CO_2_ and CH_4_ release from these different environments (Yang et al., 2016; Lara et al., 2019; Philben et al., 2020b). Flux measurements from Arctic tundra have shown that both LCPs and HCPs release CO_2_, and that LCPs tend to release more CH_4_ (often with more measurement variability) than HCPs (Wainwright et al., 2015; Vaughn et al., 2016; Arora et al., 2019). Due to their higher water content and more effective thermal insulation during cold season transitions, LCPs have been extensively studied to evaluate trends in CO_2_ and CH_4_ release (Zona et al., 2011; Roy Chowdhury et al., 2015; Tang et al., 2016). Though HCPs cover less area and may release less CO_2_ and CH_4_ than LCPs during warming (Morrissey and Livingston, 1992; Sachs et al., 2010; Wainwright et al., 2015; Vaughn et al., 2016), quantitative data for CO_2_ and CH_4_ release from HCPs is necessary to inform microbial SOC mineralization rates across tundra microtopographies in predictive climate models (Wang et al., 2019; Zheng et al., 2019). The purpose of the present study is to fill a data gap concerning the magnitude, rate, and temporal and spatial variability of warming-induced microbial SOC mineralization across HCP microtopographies and associated gradients in hydrology, and make comparisons to trends observed with LCPs.

The present study considers the biogeochemical and hydrological gradients across HCP microtopographies (troughs and centers) along a soil profile (from organic to mineral soils in the active layer), and quantifies temporal trends in C release. Our research questions include: (1) will all HCP soils – trough, center, organic, and mineral – release more CO_2_ and CH_4_ at elevated temperatures? (2) will the rates, magnitude, and composition of CO_2_ and CH_4_ release from HCP soils depend on hydraulic or geochemical factors, or both? and (3) will differences in HCP soil hydrological and thermal regimes, associated with HCP microtopographies, regulate methanogen abundance and thus CH_4_ release?

These questions were evaluated using soil incubations from cores obtained from the Barrow Environmental Observatory (BEO). First, the geochemical and hydraulic properties of HCP soils were characterized to assign soil types as either organic or mineral. Then soils were incubated at three temperatures to evaluate changes in the rate and magnitudes of CO_2_ and CH_4_ release. DNA extracted from the soils was evaluated for presence of *mcrA* genes and used to interpret CH_4_ release trends. Relationships between CO_2_ and CH_4_ release, hydraulic, and geochemical properties, and methanogen population across microtopographies and soil types were then assessed. Finally, CO_2_ and CH_4_ release, methanogen abundance, and water retention from HCPs were compared to LCP soil incubation results from Roy Chowdhury et al. (2015). The results from this study provide process understanding to field-scale observations of CO_2_ and CH_4_ fluxes in the Arctic (Sturtevant et al., 2012; Vaughn et al., 2016; Zona et al., 2016; Raz-Yaseef et al., 2017). Relative rates of gas production from these incubations complement separate field measurements to inform parameterization of Earth systems models for Arctic ecosystems (Riley et al., 2011; Burrows et al., 2020).

## Materials and Methods

### Site Description, Soil Processing, and Soil Analysis

The study site is located in the BEO in Utqiagvik, Alaska at the intensive study site B of the Next-Generation Ecosystem Experiments (NGEE) in the Arctic project (formerly Zone 1, as presented by Hubbard et al. (2013)). Located in the Arctic coastal tundra ecoregion, the site is dominated by thaw lakes and ice wedge polygons overlying continuous permafrost (Lara et al., 2015; Wainwright et al., 2015). At this site, the HCP included two microtopographic features as depicted in Figure 1: a drained, elevated center and a trough that is frequently water saturated (HCP photo, Supplementary Figure S1A). Subsurface temperatures from 2013 to 2014 at the NGEE Barrow site B varied seasonally from −18 to −1°C at 1 m below the surface^1^. Temperatures at the HCP center (0 – 25 cm) closely followed changes in air temperatures, while HCP trough temperatures varied less than the air temperature.

The core collection, soil processing, and microcosm construction methods were described previously (Roy Chowdhury et al., 2015; Yang et al., 2016). In brief, intact frozen soil cores (8.3 cm diameter) were collected in April of 2012 and 2013 using a hydraulic drill to a depth of ∼80 cm. Sample locations included the HCP center (cores NGADG0043 and NGADG0020) at N 71° 16.7583′, W 156° 36.2729′ and N 71° 16.7586′, W 156° 36.2772′, as well as the HCP trough (core NGADG0048) at N 71° 16.7576′, W 156° 36.2846′. After frozen cores were transported to Oak Ridge, TN, United States they were stored at −20°C prior to processing.

Soil cores were removed from their sterile liners and processed inside a vinyl anaerobic chamber with a N_2_ atmosphere containing at least 1.0% H_2_ and less than 1 ppm O_2_. A multi-purpose oscillating power tool with sterilized cutting blades was used for all sediment processing and homogenization in autoclaved containers kept on ice packs. Soil cores from each site were divided into 10 cm vertical increments, which were characterized for gravimetric water content (θ_*g*_), pH, and Fe(II). Fe(II) was used as an indication of redox conditions in the soil. The Fe(II) measurements are exchangeable Fe(II), extracted with 2 M KCl (1:5, w:v) by shaking for 1 h under anoxic conditions, and then filtering through 0.2 μm nylon syringe filters. Water content was determined by oven drying at 105°C for 24 h, pH using a 1:5 or 1:10 (soil weight: 2 M KCl volume) ratio, and Fe(II) with a 1,10-phenanothroline assay. Detailed methods were presented in Roy Chowdhury et al. (2015). Munsell color was documented, and soil horizons were determined per United States Department of Agriculture taxonomy.

Replicate incubations were made possible by homogenizing core depth increments with similar geochemistry in a vinyl anaerobic chamber. The depth increments were designated by soil type: organic (Oi, Oe, and Oa horizons), mineral (E and B horizons), or permafrost/ground ice (Table 1). The homogenized depth increments for microcosm incubations are as follows: HCP center organic (10–20 cm), HCP center mineral (20–50 cm), HCP trough organic (10–30 cm), and HCP trough mineral (30–50 cm). Frozen soils were homogenized using an oscillating power tool, and gravels and coarse roots were removed. This method does not affect the soil microaggregate structure or expose the samples to oxidation, drying or significant warming that could disrupt anaerobic microbial processes. Minimal oxidation occurred during this process due to handling in the anaerobic chamber. These methods are complementary to other studies that utilized less disturbed soil cubes which have more heterogeneity such as the approach of Biasi et al. (2005). Core sections from 0 to 10 cm were excluded to focus on soil processes that are not dominated by plant litter decomposition, which has been studied in many ecosystems (Hobbie and Gough, 2004; Voigt et al., 2019). Total C (TC) and nitrogen (TN) were then determined with a TruSpec CN elemental analyzer (LECO Corporation, St. Joseph, MI, United States). All measurements were conducted in triplicates and data are presented as mean ± standard deviation (Table 1). Soil preparation and experimental design are summarized in Supplementary Figure S2. Separate cores used for water potential measurements were also characterized in 10–cm increments (Supplementary Table S2) and additionally evaluated for soil texture (by hydrometer with 0.5% sodium hexametaphosphate solution) and mineralogy with X-ray diffraction (XRD) using an X’pert PRO (PANalytical, Natick, MA, United States) with Mo-Kα radiation at 55kV/40 mA, 5 – 35^*o*^ 2θ with 1.5^ o^ 2θ min^–1^ scanning rate.

**TABLE 1:** Soil characteristics of high-center polygon (HCP) center and trough cores.

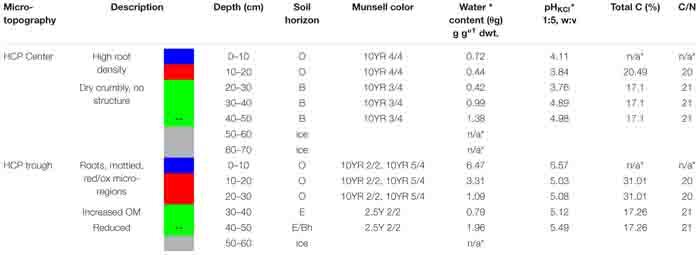

Depths determined to be plant material or litter, organic layer, mineral layer, and ice are indicated by the blue, red, green, and gray sections of the color bar, respectively. Black dashed lines within the color bar indicate the approximate depth of the active layer – permafrost boundary as determined by maximum thaw depths measured in September 2012. *Values are an average of duplicate measurements.

### Soil Water Potential Analysis

#### Soil Water Potential Measurements

Soil water potential measurements were conducted to evaluate the water retention properties of the HCP center soils collected from BEO. These measurements and parameterized models give insight into how soil water content responds to changing hydrological conditions and future trends in water availability. HCP center soils were measured for soil water potential because it was assumed that the center soils would be more sensitive to water availability dynamics than the saturated trough soils. Two complementary systems (METER Group, Pullman, WA, United States) were employed to measure soil water potential for HCP center soils (NGADG0020) during drying: the tensiometer based HYPROP system measured matric potential (Ψ*_*m*_*) for wet-end water potential, and the vapor pressure-based WP4C system measured a combination of Ψ*_*m*_* and osmotic potential (Ψ*_*o*_*) for dry-end water potentials. Electrical conductivity (EC) measurements (Orion 011010 cell, and Orion 115A+ meter) were used to adjust WP4C measurements to Ψ*_*m*_*, allowing evaluation of Ψ*_*m*_* over the full range of water contents (EC measurements at different soil: DI water ratios are presented in Supplementary Table S2). The HCP center core used for water potential measurements had slightly different depth increments associated with the soil horizons than the homogenized depths for the microcosm incubations. Depth increments for the water potential core were as follows: organic (0–10 cm and 10–20 cm), mineral (20–35 cm) with an organic rich zone at 35 cm, organic/mineral transition (35–50 cm) with indication of thaw depth at 45 cm, mineral/organic (50–65 cm), and ice (65–80 cm).

Soil core sections (at least 250 mL soil volume) were thawed and introduced to the sample rings (5 cm height by 8 cm I.D.) for HYPROP measurements (Supplementary Figure S3). Sample preparation and generation of the soil drying curve was conducted according to the manufacturer’s instructions. To account for insufficient sample volume, some HCP core sections (thawed soils from organic horizons, 0–10 cm and 10–20 cm) had to be analyzed using a reduced sample volume by adding several layers of silicon gaskets. Upper and lower tensiometers measured changes in soil water tension during drying, and the Ψ*_*m*_* and hydraulic gradient were calculated. During the HYPROP measurement, changes in mass were also recorded, allowing calculation of volumetric water content (θ_*v*_). After the air entry pressure was reached, subsamples from the HYPROP sample ring were taken from the top, middle, and bottom of the device for WP4C measurements. Additional WP4C measurements utilized air-dried samples from frozen cores. Samples lacking enough mass for WP4C measurements – namely the HCP core section from 10 to 20 cm – utilized oven-dried samples that were re-wetted. Wet end water potentials at higher θ_*v*_ were determined using the HYPROP instrument, and the dry end measurements were measured with the WP4C to produce the soil water retention curves (SWRCs), or soil water characteristic curves, which relate soil water content to soil water potential.

#### Soil Water Potential Hydraulic Modeling

Integrated water potential data from both systems were fit with five different models that are supported by the HYPROP-FIT software (version 3.0, Decagon), as described in the Supplementary Material. Model fit parameters were checked by statistical analysis of the root-mean-square error (RMSE) values of both water content data and log of conductivities and corrected Akaike Information Criterion (AICc). While five different models were evaluated, comparison of water potentials across different depth increments were made with the van Genuchten-Maulem model (Van Genuchten, 1980) (Eq. 1) because it provided the best fit for the majority of the soil depths.

(1) $$\theta_{v}\,\left(\psi \right)=\theta_{r}+\frac{\theta_{s}-\theta_{r}}{{\left(1+{\left(\alpha \,\left|\psi_{m}\right|\right)}^{n}\right)}^{1-1/n}}$$

The volumetric water content (θ_*v*_) is presented as a function of water matric potential (Ψ*_*m*_*). Fitting parameters for this study include the residual water content (θ_*r*_), saturated water content (θ_*s*_), and two shape parameters α and *n*, which represent the inverse of the air entry pressure and the soil pore size distribution, respectively.

### Incubation Experiments and Gas Analyses

Microcosm experiments were conducted in triplicate to evaluate temporal trends in the rate and magnitude of potential CO_2_ and CH_4_ production across soil horizons and microtopographies of HCPs at representative elevated temperatures (Supplementary Figure S2). Wheaton serum vials (60 mL) were filled with 15 ± 0.05 g of wet soils from organic or mineral horizons of the HCP center (NGADG0043) and trough (NGADG0048), and crimp sealed with blue butyl rubber stoppers. Replicates were from the same homogenized section of soil in the same core. Based on the soil redox condition as informed by the Fe(II) concentrations, microcosms constructed from HCP trough organic and mineral horizon soils were incubated under anoxic conditions through purging the headspace with N_2_. Mineral horizon soils from the HCP center were also incubated under anoxic conditions. The low concentrations of Fe(II) in the organic horizon of the HCP center (0 – 25 cm) indicated oxic conditions [Fe(II) data presented in Results section], and these microcosms were consequently incubated under an initial headspace of atmospheric oxygen to simulate *in situ* conditions. Headspace oxygen concentrations were monitored over time in a subset of these oxic samples (data not shown). Temperatures chosen for the incubations (−2, +4, and +8°C) were informed by the approximate thaw temperature of permafrost soils (−2°C) and the approximate maximum temperatures measured at 10 cm (+8°C) and 20 cm (+4°C) depths in HCP trough and center soils in Barrow during the summer of 2013^2^. Due to thermal gradients in the active layer, mineral soils below 20 cm depth are unlikely to experience temperatures as high as +8°C; mineral soil incubations at this temperature were conducted for comparison to organic soils, and implications of this comparison are noted in the discussion. Microcosms were destructively sampled after 0, 30, and either 60 or 75 days of incubation for analyses of pH and Fe(II) and for DNA extraction. The final sampling point (60 or 75 days) depended on the rates of CO_2_ or CH_4_ production. The total number of microcosms constructed from HCP samples was 108: 2 microtopographic features (center and trough) × 2 soil horizons (organic and mineral) × 3 temperatures (−2, +4, and +8°C) × 3 time points (0, 30, and either 60 or 75 days) × 3 replicates.

Headspace CO_2_ and CH_4_ concentrations during incubations were analyzed every other day for 10 days and, thereafter, every 5 days for up to 60 days. Gases were measured using a gas chromatograph equipped with a methanizer and flame ionization detector by injecting 0.5 mL of headspace into the GC inlet. Headspace pressure remained approximately 1 atm during incubation experiments, and adjustments for pressure changes were not made due to sampling of a small headspace volume. Total gas concentrations were reported as μmol g^–1^ dry weight (dwt.) soil and include dissolved gas concentrations, calculated as reported previously (Roy Chowdhury et al., 2015). Headspace O_2_ concentrations were monitored for microcosms from the HCP center using a SRI 8610C gas chromatograph with a 10-port gas sampling valve, argon carrier gas, 30-foot HayeSep DB 100–120 mesh column, and a thermal conductivity detector.

Concentrations (μmol g-dwt^–1^ soil) of CO_2_ and CH_4_ produced over time were fit with either hyperbolic, sigmoidal, or exponential response curves. Equations utilized for the fitting are presented in the captions of Supplementary Tables S4, S5, and are explained in detail in Roy Chowdhury et al. (2015). Rates of gas production were calculated using first derivatives of the best response curve-fitting equation at all time points to represent the changing rates of CO_2_ and CH_4_ production over time. While measurements of CO_2_ and CH_4_ release in incubations are valuable to improve understanding of relevant biogeochemical processes that occur in Arctic soils, it is important to integrate understanding developed from incubations with other experimental approaches and field scale flux measurements to effectively parameterize soil biogeochemical models. Rates presented from homogenized incubations in this study give insight into the relative importance of processes across microtopographies and soil types but are not a direct indication of net fluxes at the soil surface in the field.

### DNA Extraction and Quantitative PCR (*q*PCR) Amplification of *mcrA* Gene

Gene copies of methyl coenzyme reductase M α subunit, *mcrA*, were used to determine the presence of methanogens and further interpret CH_4_ production observed in the microcosms. DNA was extracted from subsamples of homogenized frozen bulk soil (pre-incubation) and microcosms incubated at −2, 4, and 8°C (after 30 and either 60 or 75 days of incubation). The total DNA was extracted from 0.25 g of wet soil using the PowerLyzer PowerSoil DNA Isolation Kit (MoBio Laboratories) according to the manufacturer’s protocol. DNA quality were assessed (Nanodrop2000, Thermo Scientific) and concentrations determined by Qubit 3.0 Fluorometer (Life Technologies) using the Qubit dsDNA High Sensitivity Assay Kit (Invitrogen). Soil samples containing high concentrations of organic matter and humic acids generally resulted in low purity DNA (A_260_/A_280_ < 1.5). These samples were further purified using the Wizard DNA Clean-Up System (Promega) following the manufacturer’s protocol with sample recovery ranging from 70 to 98% yield. Only samples with high-purity DNA (A_260_/A_280_ > 1.7) were used for downstream applications. Triplicate DNA extractions were performed, and samples were frozen at −20°C until quantitative PCR (*q*PCR) analysis.

The *q*PCR was performed with primers *mlas* (5′ GGT GGT GTM GGD TTC ACM CAR TA) and *mcrA-rev* (5′ CGT TCA TBG CGT AGT TVG GRT AGT) using the non-specific fluorophore iQ SYBR Green SuperMix (BioRad Laboratories Inc.) (Luton et al., 2002). All *q*PCR assays were performed using a Bio-Rad iCycler per the method described in Supplementary Table S1. Reaction mixtures contained 19 μL of the master mix and 1 μL of template DNA per reaction. Triplicates of no-template controls, containing diethylpyrocarbonate (DEPC)-treated water were included in each run. In preliminary experiments the annealing temperatures of all reactions were optimized for high-specificity and high yield when amplifying the samples. After each *q*PCR run, melt curve analysis to verify the presence of the desired amplicon was performed by increasing the temperature from 60°C to 95°C in 0.5°C increments every 5 s.

The concentration of *mcrA* gene (*C*_*target*_ [copies μL^–1^]) in the DNA standard, *Methanococcus maripaludis* C5 genomic DNA, was calculated from the DNA concentration (*C*_*DNA*_[ng μL^–1^] = 6.1 of the standard), the length of the DNA standard (*l*_*DNA*_[bp] = 1,661,137), the number of targets per DNA fragment (*n*_*target*_[copies] = 1 *mcrA* copy per genome), Avogadro’s number (*N*_*A*_) (6.022 × 10^23^ bp mol^–1^), and the average molar mass of a double-stranded base pair (*M*_*bp*_ = 660 g mol^–1^) using Eq. 2 (Brankatschk et al., 2012). The *C*_*target*_ value for *M. maripaludis* C5 was 3.3 × 10^6^ *mcrA* copies μL^–1^.

(2) $$C_{t\,a\,r\,g\,e\,t}=n_{t\,a\,r\,g\,e\,t}\times \frac{C_{D\,N\,A}\times N_{A}}{l_{D\,N\,A}\times M_{b\,p}}$$

Two separate strategies were employed to estimate the efficiency (*E*) of *q*PCR amplification of soil DNA samples based on the number of cycles necessary to reach the threshold fluorescence values (*C*_*T*_). These methods estimated either the efficiency from a standard dilution series (*E*_*ds*_) or the efficiency based on fluorescence increase (*E*_*fi*_) for each sample, as described below.

The first approach was the frequently used standard curve (SC) method of absolute quantification wherein the linear regression of log(*N*_0 standard_) versus *C*_*T*_ gives the intercept *a* and slope *b* of the standard curve (Eq. 3). The number of copies in the sample, *N*_0 sample_, can be calculated based on the linear regression of a 10-fold dilution series (*ds*) of the standard. Using this method, the limit of quantitation of *mcrA* gene concentration was estimated to be 10^2^ copies μL^–1^ (*C*_*T*_ = 29, C.V. = 0.011). The slope *b* of the linear regression (*r*^2^ > 0.999) was used to estimate the efficiency from dilution series, *E*_*ds*_ (Eq. 4).

(3) $$C_{T\,s\,a\,m\,p\,l\,e}=a+b\times l\,o\,g\,\left(N_{0\,s\,t\,a\,n\,d\,a\,r\,d}\right)$$

(4) $$E_{d\,s}=10^{\left(\frac{1}{b}\right)}$$

Using this method, the PCR amplification efficiency for all runs was determined at 98 – 101% (*E*_*ds*_ = 1.98 to 2.01). This method assumes *E*_*ds*_ of the sample is the same as that of the standard, thus introducing the possibility of increased quantification errors.

Raw fluorescence data were exported from the Bio-Rad iCycler system and imported into the LinRegPCR program (version 2014.1) (Ruijter et al., 2009). In the program settings, all samples in one *q*PCR run were treated as one amplicon to set a common window of linearity (*r*^2^ > 0.999). Subsequently, the program automatically determined the fluorescence threshold for all samples and calculated the individual *C*_*T*_ and efficiencies based on the fluorescence increase, *E*_*fi*_ (Ruijter et al., 2009). The results were exported, and the mean *E*_*fi*_ of each sample was calculated as the arithmetic mean of all replicates. The range of PCR amplification efficiency for individual *q*PCR reactions using this method ranged from 70 to 104% (*E_*fi*_* = 1.73 to 2.04). This broad range demonstrates the variability of *q*PCR efficiency, in other words in amplification quality, with template source (e.g., organic vs. mineral soils). The sub-optimum efficiency reported here may be due to the degenerate primers used to account for the *mcrA* sequence variability within the methanogen lineage, or the presence of inhibitors that can affect annealing kinetics and the accuracy of the *q*PCR assay. We used this method to obtain the *C*_*T*_ values for samples based on the linear increase of fluorescence to account for template-related variability of *E* by correcting for differences in *E* between the samples and standard (Brankatschk et al., 2012).

### Statistical Analyses

Descriptive statistics utilized for data analysis were computed in Origin Pro (version 8.6, Origin Lab). Mean comparisons were performed using Tukey’s honest significant difference *post hoc* test or the non-parametric Kolmogorov-Smirnov Test. Data are presented as mean ± standard error unless otherwise noted. Analysis of variance used Welch’s *t*-test and most statistical tests were done using the R statistical programming language (R Core Team, version 3.0.3). Non-linear regressions were performed using curve-fitting routines in Prism (version 8.4.3, GraphPad Software) as described previously (Roy Chowdhury et al., 2015).

## Results

### Geochemical Characteristics of Intact HCP Cores

Key soil characteristics of the HCP cores obtained from the center and trough were measured in 10 -cm increments along the vertical profiles. The results for the cores used in the incubation experiments are summarized in Table 1. Both center and trough cores had high contents of undegraded roots to depths of ∼10 cm, underlain by a composite of intermediately to completely degraded organic matter. The organic horizon ranged from ∼10–20 cm in the center core and from ∼10–30 cm in the trough core, indicating a deeper organic horizon in the HCP trough. Organic horizons for both center and trough cores were underlain by the mineral horizon (E and B). The HCP center core showed an extended gradient from organic to mineral horizon across depth increments of 20–40 cm. Tile probe measurements from the same HCP, obtained in a September 2012 field campaign (when the active layer was completely thawed), determined thaw depths of 40 cm in the trough and 45 cm in the center, indicating the approximate extent of the active layer, which was comparable to other reports (Wainwright et al., 2015). Permafrost in both cores was mainly ground ice. XRD data from the HCP center showed that all depths were dominated by quartz minerals with additional peaks representing albite, illite, and kaolinite (Supplementary Figure S4), expanding upon previous descriptions (Black, 1964). No inorganic carbonate minerals were observed in the XRD patterns, indicating that carbonate mineral precipitation was absent in the HCP center soil. The lack of inorganic carbonate minerals was expected based on the relatively low pH measured in these soils (Table 1). Soil textures in the HCP center were predominantly clay loam and silt loam with sandy clay in some sections (Supplementary Table S2).

The gravimetric water contents (θ_*g*_) of HCP soils increased with depth from the organic to mineral soils and were highest near the active layer-permafrost boundary. Based on volumetric water contents and soil water release measurements presented below, the HCP center active layer soils were saturated in the frozen cores. In the HCP trough, θ_*g*_ decreased with depth from the organic to mineral soils, and then increased at the active layer–permafrost transition. Profiles of θ_*g*_ variation with depth are presented in Supplementary Figure S1. The HCP trough showed greater variation in water content across the core profile (0.79 g g-dwt.^–1^ to 6.47 g g-dwt.^–1^ than the HCP center (0.42 g g-dwt.^–1^ to 1.38 g g-dwt^–1^). The organic horizon in the HCP trough had high θ_*g*_, with evidence of gleying, and high organic content (31% total C). The mineral layer of the HCP trough (17% total C) was very dark brown (Munsell 2.5Y 2/2) at depths of approximately 30 cm with visual characteristics of hydric soil. Both the center and trough of the HCP were underlain by ground ice with low mineral content. Although the C:N ratios in the active layers did not differ significantly between the center and trough, the trough had a higher % C in the organic soils indicating that there was also a higher nitrogen concentration in the trough organic soil as compared to the center organic soil. While all soil horizons had very high organic C, the trend in organic C concentration from high to low was trough organic > center organic > trough mineral ≈ center mineral.

**FIGURE 2:**
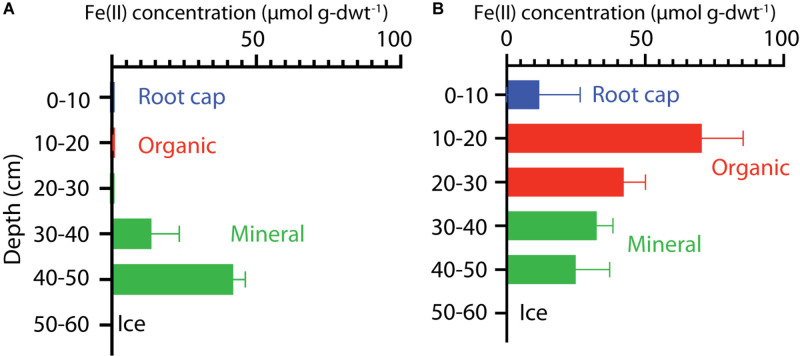
Vertical profiles of extractable Fe(II) concentrations in soil cores from HCP center **(A)** and trough **(B)**. Bars represent data from triplicate measurements with error bars showing the standard deviations. Colors indicate the different soil layers.

The extractable Fe(II) concentrations indicated a transition in redox conditions over the HCP polygon that corresponded with increasing water content. Fe(II) concentrations are plotted with depth for center and trough cores in Figure 2. The HCP center organic soil had minimal extractable Fe(II), indicating oxic conditions, and the mineral soils from 40 to 50 cm had Fe(II) at 42 ± 4 μmol g-dwt.^–1^ Fe(II) (Figure 2A). Trough organic soils had even higher Fe(II) at 70 ± 15 μmol g-dwt.^–1^, a very significant increase from the undetectable Fe(II) in the HCP center organic soils. Decreases in Fe(II) were observed with increasing depth from the HCP trough organic to mineral soils (Figure 2B). The high Fe(II) concentration observed in the HCP trough organic samples aligned with elevated θ_*g*_, indicating that the saturated organic horizon of the trough was more anoxic than the drier organic horizon in the HCP center. Trends in Fe(II) concentrations mirrored the general trends in θ_*g*_ across microtopographies and horizons, highlighting the positive relationship between Fe(II) and θ_*g*_. However, the high volumetric water content of the HCP center organic soils with low concentrations of extractable Fe(II) demonstrates that the water content does not always indicate anoxic conditions. Overall, the Fe(II) data demonstrates a gradient from more oxidized to more reduced conditions across the relatively oxic/sub-oxic surface layers in the HCP center to the anoxic HCP trough.

Higher Fe(II) concentrations were also generally associated with higher pH. HCP trough samples had significantly higher pH than the HCP center samples (*p* < 0.05, two-tailed paired *t*-test), with the pH of the trough and center samples across all depths averaging 5.26 and 4.32, respectively. Lower pH in shallower HCP center soils (Table 1) may represent acidity produced during oxidation of Fe(II) and precipitation of Fe(III) oxide/hydroxides or anaerobic SOC decomposition. The lower pH in HCP center soils could also be related to decreased buffering capacity associated with lower cation concentrations in HCP center soils (Newman et al., 2015).

### Soil Water Potential and Hydraulic Modeling

Soil water potential indicates how tightly water is held in a soil matrix. Measurements of soil water potential were conducted using an additional HCP center core and were used to interpret the water retention capacity of the soil (Moon and Graham, 2017). The water retention of tundra soils may have important implications for future trends in SOC mineralization, which are discussed in Section “Carbon Release From HCPs May be Associated With Higher Water Content and Total C.” Soils from the HCP center were evaluated because they are expected to have more dynamic wetting and drying cycles than the mostly saturated HCP trough. Soil properties for the additional core were determined using the same techniques as the microcosm incubation cores and are presented in Supplementary Table S2. Electrical conductivity (EC) was also determined at different dilutions before collecting water potential data (Supplementary Table S2).

#### Properties Used for the HYPROP and WP4C Soil Water Measurement

The HCP center core used for water potential measurements (NGADG0020) had pH ranges (3.88 – 4.46) comparable to the core used for microcosm incubations (3.76 – 4.89). Total C between cores was also similar: the water potential core had 11–19% (Supplementary Table S2) total C and the microcosm core had ∼17–20% total C (Table 1). Well-decomposed organic layers in the 35–50 and 65–80 cm sections were also observed. The EC values for the HCP core exhibited a proportional dilution effect with increased water to soil ratio for most depth increments, however, the 30–50 cm and 65–80 cm sections from HCP showed similar or higher EC values with increased water volume (Supplementary Table S2). HCP center core depths that did not have proportional dilution for EC also had higher gravimetric water contents.

#### Model Fitting to Determine Retention and Conductivity Function Parameters

**FIGURE 3:**
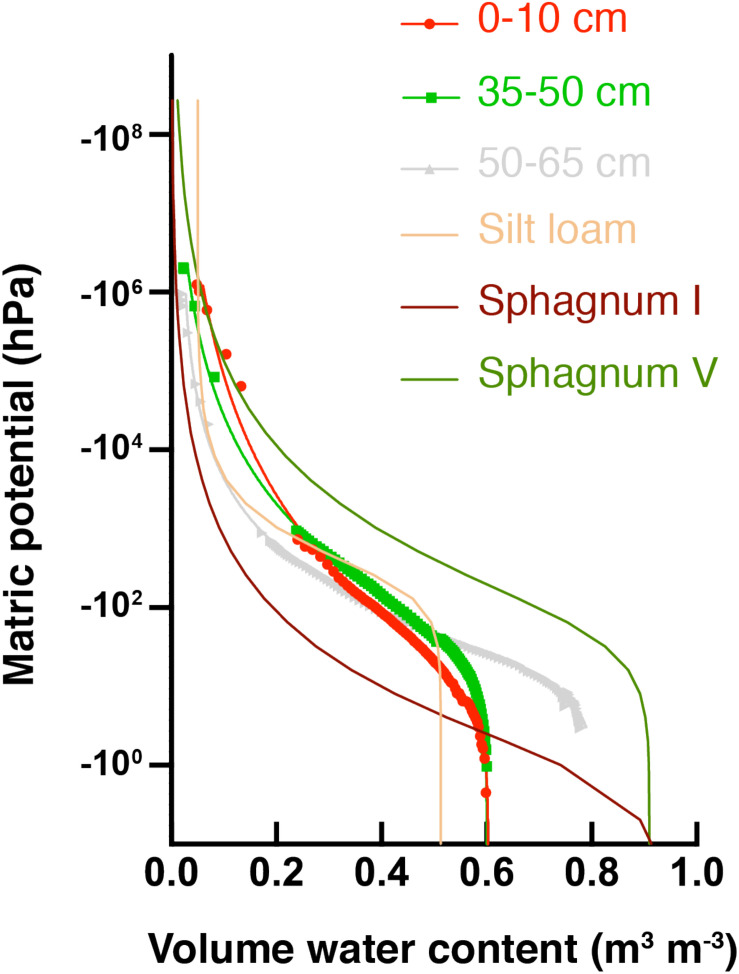
Soil water retention curves (SWRCs) for HCP center at different soil depths (red circles = organic, green squares = mineral, and gray triangles = permafrost). Parameters for reference curves for peat soils are from Liu and Lennartz (2019): group I sphagnum had a low bulk density (BD), while group V had a relatively high bulk density. Parameters for silt loam are from Or et al. (2012). Saturated water contents (θ_*sat*_) are the volumetric water content (θ_*v*_) at the highest Ψ_*m*_.

Water potential measurements across different soil depths were used to develop SWRCs and the data was fit with the van Genuchten model (Figure 3). Solid lines associated with data points in Figure 3 are the best fit SWRCs for three selected soil depths. Fitting parameters are presented in Supplementary Table S3, and additional representations in terms of hydraulic conductivity are included in Supplementary Figure S7. All soil depths from the HCP center core had higher saturated water contents (θ_*sat*_) than a common silt loam (tan line in Figure 3), but lower θ_*sat*_ than typical sphagnum peat soil. Saturated water contents determined from HCP soil model fitting (θ_*s*_ in Supplementary Table S3) were similar in the different soil sections (∼60–65%), except for the 50–65 cm and 65–80 cm depths which were greater (81 and 74%, respectively). These higher saturated water contents are likely due to particularly high porosity resulting from ice formation near the active layer – permafrost boundary.

Macroporosity of soils can be estimated from the percent pore volume extracted (beginning with saturation) under a fixed value of Ψ_*m*_ (Beven and Germann, 1982). For the present analysis, –10 hPa Ψ_*m*_ was used to evaluate macroporosity (Supplementary Figure S8). The macroporosity for the deepest soil layer, 65–80 cm, was quite high (35%), consistent with the interpretation that ice formed large pores at this depth. The macroporosity of the 0–10 cm depth was 13%, which is still high relative to high bulk density (BD) sphagnum soils (2.6%). Water drains quickly through larger pores in HCP center soils, but active layer organic soils (depths of 0–10 cm and 10–20 cm) show that they will retain more water under continued drying than thawed permafrost soils (depths of 50–65 cm and 65–80 cm), as indicated by the higher θ_*v*_ at lower Ψ_*m*_ for organic soils.

Other fitting parameters from the van Genuchten-Mualem model (Supplementary Table S3) give further insight into the hydraulic behavior of the HCP center soils. Soils from depths 50–65 cm and 65–80 cm had high porosity (>0.8) and high saturated hydraulic conductivity (K_*s*_), and an increase in K_*s*_ (from ∼10^1^ to ∼10^4^cm day^–1^) was observed from top to bottom of the soil core. The shape parameter, α, shows an increase with depth for the HCP center. Elevated α values are often associated with less water retention. The data indicate that the soils near the active layer – permafrost boundary would poorly retain water. These measurements describe soil properties from a newly thawed soil core, and do not account for future compaction or subsidence that could reduce porosity and conductivity in the field.

### CO_2_ and CH_4_ Production From HCP Incubations

HCP soils were incubated at −2, +4, and +8°C. All incubations were initially anoxic (headspace purged with N_2_), except for the HCP center organic horizon incubations (initially with atmospheric oxygen). CO_2_ and CH_4_ were measured for at least 50 days, sampling every other day for 10 days and thereafter every 5 days, and high frequency temporal measurements of CO_2_ and CH_4_ allowed for response curve fitting.

#### Magnitude and Character of CO_2_ Release Varies With Soil Type and Microtopography

**FIGURE 4:**
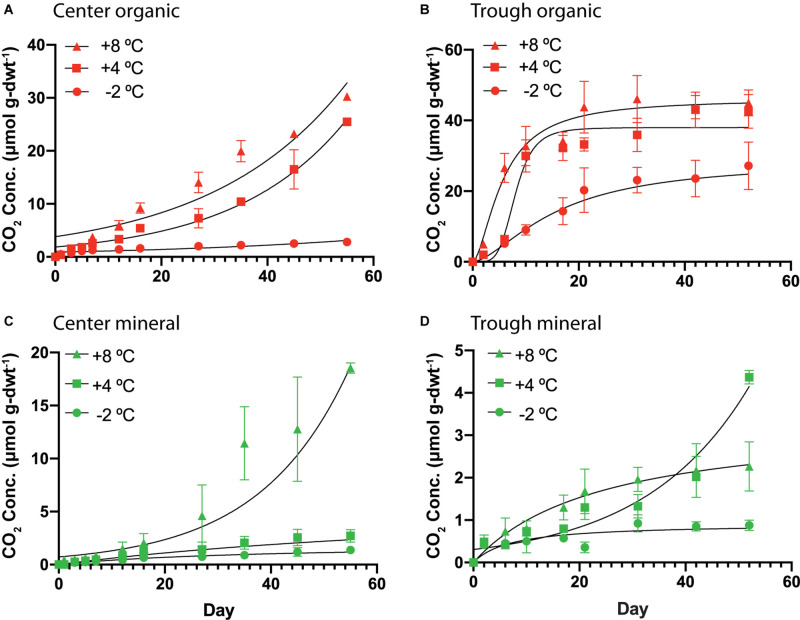
Cumulative CO_2_ production per mass of soil as dry weight (dwt.) added to the incubations over time from HCP center organic **(A)**, trough organic **(B)**, center mineral **(C)**, and trough mineral **(D)** soil samples (*n* = 3) incubated at –2, 4, and 8°C. The error bars represent standard deviation. Curve-fitting equations for each condition were determined following procedures described previously (Roy Chowdhury et al., 2015), and parameters are listed in Supplementary Table S4. Vertical scales are different for the four different plots.

Organic and mineral soils of the HCP showed differences in the magnitude and character of CO_2_ production. Cumulative CO_2_ production over time is plotted in Figure 4 for organic and mineral horizons of the HCP center and trough, and higher temperatures generally resulted in elevated CO_2_ production under all conditions. Parameters used to fit the response curves are presented in Supplementary Table S4. In the HCP center soils (Figure 4A), oxygen was consumed within the first 13–15 days of incubation for microcosms at +4 and +8°C (data not shown); those incubated at −2°C were not analyzed. As expected, CO_2_ production in the HCP center and trough organic soils increased at elevated temperatures (Figures 4A,B). The rates of CO_2_ production (Supplementary Figure S5) increased at later times (>20 days) for all temperatures in the HCP center organic soils, which contrasts with the initial increased rates of CO_2_ release which peaked and then decreased at later times in HCP trough soils (except for the trough mineral soil at 4°C which showed exponential response, Figure 4D). Decreasing rates of CO_2_ production at later incubation times has been previously observed for active layer soils over similar incubation times (Waldrop et al., 2010). The initial lag in CO_2_ release in the HCP center organic soils could be related to its relatively low water content which could impact initial microbial activity, though the lower pH may also be a factor. The HCP organic trough samples showed a peak in CO_2_ production rates at 2 days (∼4.9 μmol CO_2_ g-dwt^–1^d^–1^) and 9 days (5.8 μmol CO_2_ g-dwt^–1^d^–1^) of incubation at +8°C and +4°C, respectively (Supplementary Figure S5), which could indicate more efficient utilization of more bioavailable C with increasing temperature in the saturated trough organic soils (Yang et al., 2016).

In general, the mineral horizons of the HCP center and trough demonstrated lower cumulative CO_2_ production than the organic horizons over the experimental time. At +8°C, mineral soils of the HCP center (Figure 4C) showed exponential response, with accelerating CO_2_ production. This is especially clear in Supplementary Figure S5, which presents the rate of CO_2_ production as a function of time. While HCP organic soils generally showed higher cumulative CO_2_ production, mineral soils from HCPs could still be contributing to CO_2_ release in Arctic permafrost regions, albeit at lower rates.

#### HCP Trough Soils May Be a Significant Source of CH_4_

**FIGURE 5:**
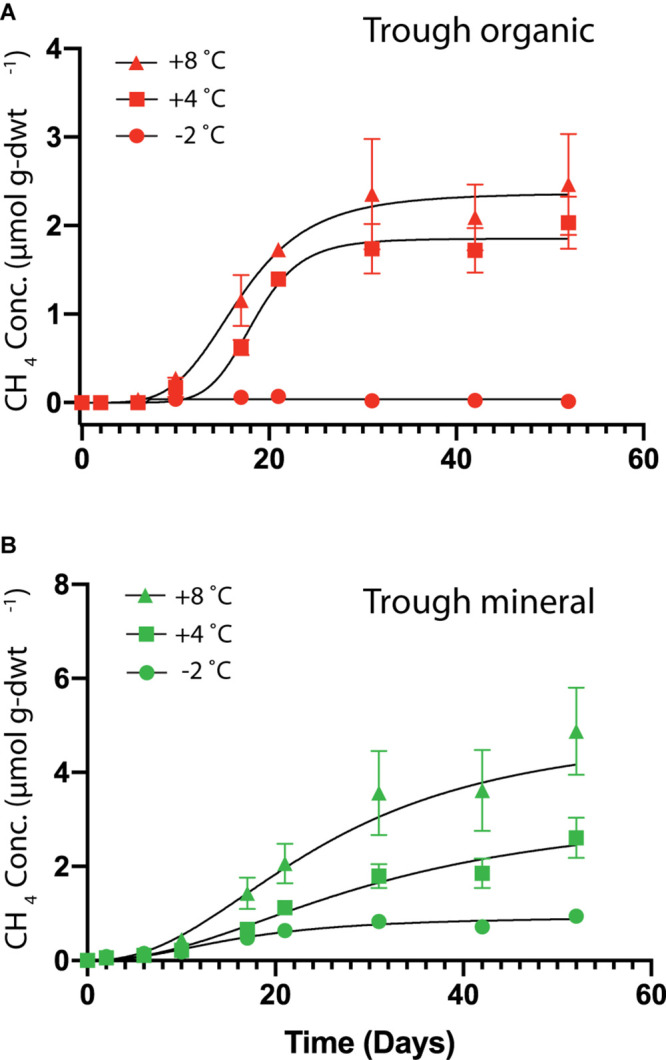
Cumulative CH_4_ production per mass of soil as dry weight (dwt.) added to the incubations over time from HCP trough organic **(A)** and trough mineral **(B)** soil samples (*n* = 3) incubated at –2, +4, and +8°C. No CH_4_ production was observed from the HCP center soil incubations. The error bars represent standard deviations. Curve-fitting equations for each condition were determined following procedures described previously (Roy Chowdhury et al., 2015), and parameters are listed in Supplementary Table S5. Note: Vertical axes are different scales for the two different plots.

HCP trough soils demonstrated elevated cumulative CH_4_ production at higher temperatures, while the HCP center soils of both the organic and mineral horizons did not show measurable CH_4_ production over 50 days. Figure 5 presents cumulative CH_4_ production over time for HCP trough soils, with the mineral horizon releasing approximately twice as much CH_4_ (5 μmol g-dwt.^–1^) as the organic horizon (2.5 μmol g-dwt.^–1^) at 8°C over 50 days. CH_4_ production plateaued in the trough organic soil after 30 days (Figure 5A) but did not reach a plateau in the trough mineral soil even after 50 days (Figure 5B). Rates of CH_4_ production, which are plotted against time in Supplementary Figure S6, peak between 5 and 20 days for all conditions, indicating that delayed methanogen activity may have been followed by a phase of substrate limitation. Previous incubations by Zona et al. (2012) with similar soil types also indicated a peak in methane production rates, though this was observed slightly later at 30 days of incubation. Overall, most of the C lost from the HCP soils was released as CO_2_ rather than CH_4._ However, the global warming potential of CH_4_ is substantially higher than CO_2_, and greenhouse gas emissions from HCP troughs may be underappreciated at present. Production of CH_4_ in the mineral horizon of the HCP trough accounted for 67% of total C loss at +8°C, while CH_4_ released from the organic horizon approximated ∼ 5% at +8°C. Methanogenesis at −2°C was negligible throughout.

### *mcrA* Detected in Methane Producing Soils

**FIGURE 6:**
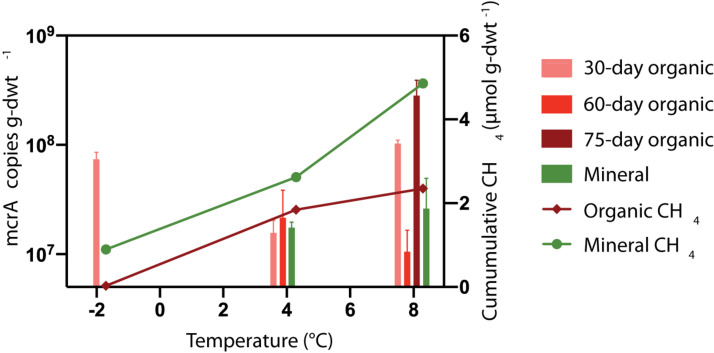
Mean temperature responses of *mcrA* copy numbers (*n* = 3) for HCP trough soils. DNA was extracted for qPCR analysis after incubating organic soils for 30 and 60 or 75 days. Mineral soils were incubated for 30 and 60 days (8°C) or 75 days (4°C). The *mcrA* gene was not detected (<10^2^ copies μL^− 1^) in HCP center soils. Mineral soils and organic soils incubated 60 days at –2°C were not analyzed.

It was expected that microcosms with elevated CH_4_ production would also have larger methanogen populations. The *mcrA* abundance was quantified in microcosms after 30, 60, and/or 75 days of incubation at −2, +4 and +8°C. Figure 6 presents the *mcrA* copy numbers for HCP trough soils. No amplifiable signature for *mcrA* could be detected in the HCP center soils (<10^2^ copies g-dwt.^–1^ soil), which was consistent with the lack of CH_4_ produced. This gene also was undetected in frozen HCP trough samples before incubation, which could indicate a low methanogen population or inhibitory substances in the soil. Overall, *mcrA* copy numbers for HCP trough soils were high (10^7^ to 10^9^ copies g-dwt^–1^ soil) after incubation, but trends in CH_4_ release between layers or across incubation temperatures were not observed (Figure 6).

## Discussion

### Variation in C Release Across HCP and LCP Microtopographies and Soil Types

**FIGURE 7:**
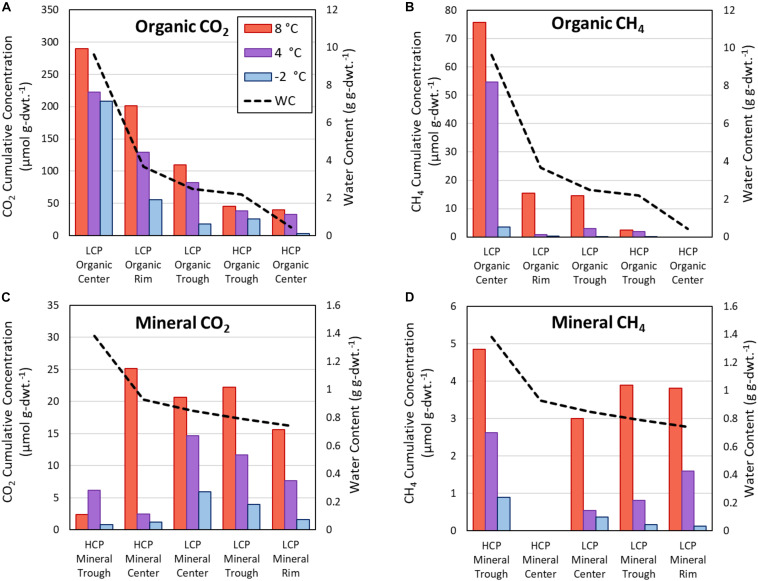
Cumulative CO_2_ and CH_4_ release from active layer samples after incubations as determined from response curves at 60 days. Carbon release from organic soils for CO_2_ **(A)** and CH_4_ **(B)**, as well as mineral soils for CO_2_ **(C)**, and CH_4_ **(D)**. The vertical axes scales differ for each panel. Horizontal arrangement of LCP and HCP microtopographies progresses from highest water content (left) to lowest water content (right), and the water content relationships are different for organic and mineral soils. WC, water content.

The present study focused on HCPs and demonstrated variations in magnitude and rate of CO_2_ and CH_4_ release across HCP microtopographies and soil types at different temperatures. Several previous studies have similarly quantified the magnitudes and rates of C release for LCPs during soil warming (Herndon et al., 2015; Roy Chowdhury et al., 2015; Wagner et al., 2017). Figure 7 compares trends across all three temperatures for HCP centers and troughs with previously published data (Roy Chowdhury et al., 2015) for LCP microtopographies (center, rim, and trough). The additional rim microtopography in LCPs is defined as the raised edges between the center and the trough (not present in HCPs). As expected and consistent with previous LCP incubations, elevated temperatures in HCP soil incubations resulted in greater cumulative CO_2_ release after 60 days – increasing from −2, to 4, to 8°C – for all microtopographies and soil types (Figures 7A,C), with the exception of the HCP trough mineral soils (Figure 7C). Similarly, higher cumulative CH_4_ was observed as temperatures increased for all microtopographies and soil types (Figures 7B,D).

Overall, the incubations indicated that soils from almost all microtopographic features of both LCPs and HCPs released more CO_2_ and CH_4_ with increasing temperature, which is consistent with trends in mean CO_2_ production at elevated temperatures observed across as range of anoxic incubation experiments [see synthesis in Treat et al. (2015)]. While previous anoxic incubations have used elevated temperatures (in excess of 20°C) to represent the maximum possible carbon that could be released from the soils, the temperatures of −2, 4, and 8°C were used in this study to represent soil temperatures that are more likely to occur at the tested soil depths during near future warming conditions. Relative rates of gas production from these experiments help to parameterize temperature response functions that modulate gas production functions in biogeochemical models (Riley et al., 2011). For example, Figure 4 shows that CO_2_ produced from center organic soils at 8°C is approximately 15 times greater than CO_2_ released from center mineral soils at 4°C after 60 days of incubation. Simulations using these response functions should be compared with future research that evaluates the magnitudes of CO_2_ and CH_4_ production along a soil profile with a thermal gradient representative of temperature maximums under field conditions.

Water availability appeared to impact both CH_4_ and CO_2_ production, consistent with previous observations (Gulledge and Schimel, 1998; Lipson et al., 2012; Kane et al., 2013; Moyano et al., 2013). In Figure 7, the microtopographies on the horizontal axis are arranged from highest to lowest θ_*g*_ (left to right) for organic (Figures 7A,B) and mineral (Figures 7C,D) soils. A trend showing increased CO_2_ and CH_4_ release with elevated θ_*g*_ is apparent for organic soils, but the same trend is not observed for the mineral soils, which have a narrower range of θ_*g*_ (0.74 – 1.38 g g^–1^) than the organic soils (0.44 – 9.62 g g^–1^). The relatively dry, aerobic HCP center soils had lower CO_2_ and CH_4_ flux than the more saturated LCP center soils, which is consistent with a synthesis of anoxic incubations by Treat et al. (2015) that showed lower CO_2_ and CH_4_ production from dry soils than soils that were saturated or experienced a fluctuating water table. Elevated water contents are known to limit oxygen transport in soils, restricting aerobic respiration. Ecological models commonly enforce schemes in which water saturation prohibits C mineralization and stimulates methanogenesis (Riley et al., 2011; Burrows et al., 2020), but they do not adequately account for anaerobic CO_2_ production. CO_2_ concentrations observed in this study (Figure 4) suggest that anaerobic processes are contributing CO_2_ release. It has been shown that anaerobic processes such as microbial Fe(III) reduction coupled with oxidation of small organic acids can release CO_2_ (Lovley and Phillips, 1988), and that this process can impact CO_2_ release under some soil conditions (Chen et al., 2020).

Change in the rates of CO_2_ and CH_4_ release were observed for both HCP and LCP incubations. C mineralization rates in the LCP center soils generally decreased over time, while the HCP center soils under some conditions showed increasing rates of C release at later times (Figure 4). While some incubations plateaued in C production rate after 50 days, other incubations were just beginning to increase in C production rate (Supplementary Figures S4, S5); this could indicate delays in C release due to slow growth of the heterotrophic microbial community or substrate competition. Previous soil incubations from soils of drained tundra lakes also showed an earlier time peak in CO_2_ production, and a delay in CH_4_ production (Zona et al., 2012).

Overall, the LCPs showed higher cumulative CO_2_ and CH_4_ release and more variability in the magnitude of C release than HCPs. Despite releasing less C and covering approximately half the land area as LCPs near Utqiaġvik, HCP soils can release large quantities of CH_4_ and CO_2_ when integrated across the large spatial domains of polygonal Artic tundra, and they need to be incorporated in models that estimate total C flux from Arctic soils. For example, data from Lara et al. (2015) indicate that HCPs cover ∼200 km^2^ on the Utqiagvik Peninsula alone. Flux measurements from chamber experiments indicate that both CO_2_ and CH_4_ are released from HCPs and that the amount of C release varies across seasons (Wainwright et al., 2015; Arora et al., 2019). Incubation studies such as the present study allow for precise temperature control and dense temporal measurements that aid in understanding the dynamic nature of CO_2_ and CH_4_ release from Arctic soils. Knowing the variability in relative amounts of CO_2_ and CH_4_ released from incubations and the timing of peak release rates for different soil types and microtopographies may help constrain parameters needed for models that estimate carbon flux from Arctic soils. Greater understanding of processes developed from controlled, homogenized incubations can be used to inform intact soil core incubation studies, field measurements, and development of reactive transport models, which ultimately allow for better estimates of C release from Arctic tundra soils.

### Carbon Release From HCPs May Be Associated With Higher Water Content and Total C

In addition to elevated incubation temperature, higher water content and total C across different soil types and microtopographies appeared to be related to greater cumulative CO_2_ and CH_4_ release from the HCP soils. The HCP trough organic soil, with the highest water content (1.09–3.31 g g-dwt.^–1^) and organic C (31%), showed the highest cumulative CO_2_ production over 50 days (Figure 4B) and the highest rates of CO_2_ production (Supplementary Figure S5), indicating the potential importance of anaerobic processes such as fermentation, syntrophic oxidation, acetoclastic methanogensis, and iron reduction which produce CO_2_ release under anoxic conditions. Mineral soils from both trough and center microtopographies had lower organic C, which may explain the lag and lower magnitude in CO_2_ production than the organic soil – though this observation may also be due to limitations on microbial metabolism. HCP center soils with relatively low θ_*g*_ produced no CH_4_ over 50 days, although O_2_ was effectively consumed within the first 13–15 days of incubation. On the other hand, CH_4_ was measured in both organic and mineral horizons of the HCP trough, which had higher θ_*g*_ and no initial oxygen present in the headspace (Figure 5 and Table 1). Alternatively, CH_4_ production may be controlled by the biomass of methanogens present in the soils.

### Water Potential Influence on Carbon Release From HCP and LCP Soils

Positive correlations between C release and soil water content are discussed above, but this does not necessarily indicate the longer-term potential for CO_2_ and CH_4_ flux from Arctic soils. It is becoming more widely understood that climate change trends in the Artic could result in a drier landscape, exemplified by transitions from LCPs to HCPs (Raynolds et al., 2014). It will likely be comparably important to understand the ability of soil to retain water under a drying climate as it is to know the present water content at discrete time points.

In this study, soil water retention curves constructed for HCP center soils (Figure 3), showed water retention with relatively low potentials. This indicates that HCP soils can retain substantial amounts of water during drying. For example, at a permanent wilting point of –1.5 MPa, the HCP organic and mineral layers retain more water than a typical silt loam soil, similar to decomposed sphagnum soil. These results were different from those observed in LCP soils. Soil water retention curves for the LCP center organic soil showed lower water retention at higher water potentials than the LCP center mineral soils and all depths of HCP center soils (Supplementary Figure S7). Parameter fitting indicated very high θ_*s*_ (1.0) and high hydraulic conductivity for LCP center organic soils (Supplementary Table S3). Estimates of macroporosity in LCP organic soils were significantly higher (23.9%) than underlying mineral soils (2.1%) and HCP active layer soils (Supplementary Figure S8), indicating that LCP organic soils may lose water from large pores relatively fast during dry seasons. These seasonal trends could potentially influence future ecological succession and SOC mineralization by microbial communities, which was previously proposed for soil moisture impacts on methanotrophs (Gulledge and Schimel, 1998). The large pores in these organic soils store substantial amounts of water but drain quickly from active layer soils and more slowly from thawed HCP permafrost. Previous work by Gulledge and Schimel (1998) showed faster rates of CH_4_ oxidation at water holding capacities between 30 and 50%, and indicated that CH_4_ oxidizers in wetter environments were less sensitive to changes in water potential than those in sampled in drier environments. Future studies should further explore the role of macroporosity and soil water retention in maintaining active microbial communities in Arctic tundra soils and the impact on CO_2_ and CH_4_ release.

### Controls on Methanogen Presence and CH_4_ Production

Counter to the original hypothesis, methanogen biomass estimated with *mcrA* marker gene copy numbers did not show a strong correlation with CH_4_ release magnitude or rate. *mcrA* copy numbers in HCP trough soils did not correlate with the cumulative CH_4_ production (Figure 6), and similar observations were made for LCP soils (Supplementary Figure S9). One possible explanation for this discrepancy is that the presence of *mcrA* genes does not necessarily indicate active methanogens are present; inactive or dead organisms can still carry quantifiable amounts of *mcrA* (Freitag and Prosser, 2009). Although detectable levels of *mcrA* were present in LCP trough (2.5 × 10^5^ copies g dwt.^–1^) and center (1.3 × 10^7^ copies g-dwt.^–1^) soils measured before any incubation, significant incubation time was required before methanogenesis rates peaked in most samples (Supplementary Figure S6). Future studies would benefit from measuring gene expression to determine which microorganisms and enzymes are active.

While strong correlations between copy number and CH_4_ release were not observed, the lack of detectable *mcrA* was consistent with HCP center soils that did not produce CH_4_. Though we cannot rule out soil inhibitors preventing the amplification of *mcrA*, the replicate measurements made for all HCP center soils indicate that methanogens were not present. Low methanogen abundances or methanogenesis rates have been previously reported in permafrost environments (Wagner et al., 2007; Steven et al., 2008; Waldrop et al., 2010), and HCPs had lower relative methanogen 16S rRNA gene abundance than LCPs (Taş et al., 2018). Potential reasons for the lack of methanogens in the HCP center soils include (1) competition for substrate from other microbial communities, namely iron reducers (Roden and Wetzel, 1996), (2) limited water availability, (3) intermittent oxic conditions in the field limiting anaerobic activity and measurable methanogen growth before incubations, or (4) low pH. Previous studies have documented competition for substrate between iron reducers and methanogens (Philben et al., 2020b), but the incubations in the present study did not show a significant change in Fe(II) concentrations over the incubation time (data not shown). Water contents measured in the HCP center mineral soils were only slightly lower than the water contents in the HCP trough soils, and it is assumed that this small difference was not the main control. Fe(II) concentrations indicated that HCP center soils supported iron reduction (Figure 2), but it is possible that intermittent oxic conditions prevented soil redox conditions from becoming reducing enough to support methanogenesis in HCP center soils. Other geochemical controls such as pH may also influence the spatial variability in methanogenesis. While methanogens are known to grow at pH below 5, their growth is optimal between pH 6 and pH 8 (Garcia et al., 2000). The low levels of methanogenesis and *mcrA* measured in HCP soils indicate that low CH_4_ production rather than robust CH_4_ oxidation explains low CH_4_ emissions from these elevated soil surfaces. Geochemical controls in addition to methanogen presence are likely to determine the rate and magnitude of CH_4_ release from Artic tundra soils.

### Implications for CO_2_ and CH_4_ Emissions From Warming Arctic Tundra

Knowledge of the temporal and spatial variability in microbial C mineralization rates of Arctic soils in response to warming are key to constraining uncertainties in predictive climate models. The present study focuses on CO_2_ and CH_4_ release from HCPs during soil incubations and quantifies nature and magnitude of C release using response functions. The magnitudes and rates of CO_2_ and CH_4_ production from HCPs were compared with C production from LCPs, and it was determined that the total C released from organic soils of both LCPs and HCPs is greater and more variable than the C released from mineral soils (Figure 7). Under our experimental conditions, it was also concluded that relatively more C is released from LCPs than HCPs, but the contributions from HCPs – especially the initial CH_4_ release from the more saturated, anoxic HCP troughs – are significant and should not be neglected when evaluating soil C flux (Figure 5 and Supplementary Figure S6). Anoxic soils that retain water are more likely to release CH_4_, and methanogen marker gene abundance does not always correlate with increased CH_4_ release magnitudes or rates when other geochemical controls are limiting.

Increased warming and thermokarst formation is predicted to increase the HCP coverage and decrease that of LCP in polygonal tundra landscapes (Lara et al., 2015). Our results suggest that this transition to drier HCPs may result in less C release as CH_4_ and CO_2_ from organic soils, and that releases from mineral soils should remain relatively uniform across geomorphic features. Soil warming in the present saturated landscapes and a longer thaw season can also cause an increasing positive C feedback due to accelerated loss of CO_2_ and CH_4_ (Hinzman et al., 1991), particularly due to high rates of methanogenesis at the end of thaw season (Windsor et al., 1992; Mastepanov et al., 2008; Sturtevant et al., 2012). The results from the present incubation study can be used to inform targeted field measurements of CO_2_ and CH_4_ release across soil types and microtopographies and validate relevant processes and relative rates before model implementation. Although polygon troughs make up a small proportion of the land surface, these saturated areas may contribute disproportionately to both CH_4_ and CO_2_ emissions. The maximum rate of CO_2_ production in the saturated HCP trough organic soil was more than 3× higher than in the unsaturated HCP center (Supplementary Figure S5), and CH_4_ in HCP trough soils was released at rates up to 0.19 μmol g-dwt.^–1^ day^–1^. Mechanisms promoting more CO_2_ release from the HCP trough than the HCP center, may be due to the fitness of the microbial communities, limitations on nutrient transport in drier environments, or environmental stressors such as low pH in the HCP center. Future work should target these potential mechanisms directly. While CH_4_ emissions are minor components of C loss compared to anaerobic CO_2_ emissions in Utqiaġvik (Lipson et al., 2012), this proportion of CH_4_ could vary significantly across the Arctic due to differences in soil freezing and the availability of alternative electron acceptors. Future studies evaluating CO_2_ and CH_4_ release from permafrost soils should directly evaluate the influence of soil water retention on CO_2_ and CH_4_ flux during soil drying, and fully explore temporal changes in microbial communities and metabolites with and without soil drying.

## Conclusion

High centered polygons soil incubations complemented previous LCP soil incubations to provide relative rate data for CO_2_ and CH_4_ production across the microtopographic extremes of polygonal tundra. Organic soils from LCPs produced the most greenhouse gases, while mineral soils had similar production rates regardless of microtopography or water content. Dynamic changes in these soils due to precipitation, freeze-thaw and drainage processes were magnified in organic layers, where temporary water saturation may not predict rates of anaerobic processes, including methanogenesis and iron reduction.

## Data Availability Statement

The original contributions presented in the study are included in the article/Supplementary Material, further inquiries can be directed to the corresponding author/s.

## Author Contributions

TRC, J-WM, BG, LL, SW, and DG designed the experiments. TRC performed the incubation experiments, chemical, and molecular analyses. J-WM performed XRD and water release experiments. TRC, EB, J-WM, and DG analyzed the data and wrote the manuscript. All authors edited and reviewed the manuscript.

## Conflict of Interest

The authors declare that the research was conducted in the absence of any commercial or financial relationships that could be construed as a potential conflict of interest.

## Footnotes

1 http://lapland.gi.alaska.edu/vdv/vdv_historical.php

2 http://lapland.gi.alaska.edu/vdv/vdv.php/historical/59
