# Supplementary material for: Temporal, Spatial, and Temperature Controls on Organic Carbon Mineralization and Methanogenesis in Arctic High-Centered Polygon Soils
Source: Front Microbiol. 2021 Jan 11;11:616518. doi: 10.3389/fmicb.2020.616518 (PMC7829362; doi:10.3389/fmicb.2020.616518)
Supplement: Supplementary file 1 [file Data_Sheet_1.docx]

Supplementary Information

# Supplementary Methods

**Soil water potential methods and modeling (extended Section 2.2)**

The water matric potential (Ψ_m_) represents the energy or pressure difference between free water and water bound to the soil matrix: this is a negative value except when the soil is saturated. In general, elevated θ_v_ at lower Ψ_m_ on a SWRC indicates that a soil retains more water during drying, and lower θ_v_ at higher Ψ_m_ is associated with lower saturated water content (θ_sat_), which is defined as the maximum amount of water a soil can store (maximum θ_v_ at high Ψ_m_).

Five models were applied to fit the water potential data using the HYPROP-FIT software (version 3.0, Decagon): Brooks-Corey (Brooks and Corey, 1964), Fredlund-Xing (Fredlund and Xing, 1994), Kosugi (Kosugi, 1996), van Genuchten (m=1-1/n, Mualem model), and van Genuchten (mnvar) (Van Genuchten, 1980). Four conditions were evaluated including original, Peters-Durner-Iden (PDI, variant implying the water content matches zero at oven dry), bimodal, and bimodal+PDI. Each fitting was checked with statistical analysis by root-mean-square error (RMSE) values of both water content data and log of conductivities and corrected Akaike Information Criterion (AICc).

# Supplementary Figures and Tables

## Supplementary Tables

**Table S1 |** Primers and qPCR methods for *mcrA* gene quantification.

| ***mcrA*** | | |
| --- | --- | --- |
| **Standard** |  | *Methanococcus maripaludis* C5 |
| **Forward Primer (mlas)** |  | 5′ GGT GGT GTM GGD TTC ACM CAR TA |
| **Reverse Primer (mcrA-rev)** |  | 5′ CGT TCA TBG CGT AGT TVG GRT AGT |
| **Method** | | |
| Denaturation |  | 95 ^o^C, 30 sec |
| Annealing |  | 61^o^C, 30 sec |
| Elongation |  | 72 ^o^C, 30 sec |
| Master mix composition for 96-well plate |  | 550 µL PCR grade water, 220 µL each of forward and reverse primer (5 pmol µL^-1^), 1100 µL iQ SYBR Green SuperMix |

| **Table S2 \|** Soil characteristics of HCP and LCP center core samples used for water potential measurements. | | | | | | | | | | | | | | | | | | | | | | |
| --- | --- | --- | --- | --- | --- | --- | --- | --- | --- | --- | --- | --- | --- | --- | --- | --- | --- | --- | --- | --- | --- | --- |
| Micro- Topography | Depth | Munsell Color | Soil pH |  | Electrical Conductivity  (μS cm^-1^)* | | | |  | Water |  | Water |  | C | N | C/N |  | Soil texture^3^ | | | |  |
|  | (cm) | (wet) | (1:10, w:v) |  | 1:5 | 1:10 | 1:15 | 1:20 |  | *θ_g_^1^* |  | *θ_v_^2^* |  | (%) | (%) |  |  | Clay | Silt | Sand | >2mm |  |
| HCP  Center | 0-10 | 10YR2/2 | 3.91 |  | 458 | 212 | -- | -- |  | 0.68 |  | 64.9 |  | 15 | 0.8 | 19 |  | 27 | 32 | 41 | 4.44 |  |
|  | 10-20 | 10YR 4/2 | 3.88 |  | 449 | 204 | -- | -- |  | 0.76 |  | 65.5 |  | 18 | 1 | 18 |  | 17 | 52 | 31.1 | 5.8 |  |
|  | 20-35 | 5YR 2.1/1 | 3.96 |  | 389 | 202 | -- | -- |  | 0.58 |  | 64.4 |  | 15 | 0.9 | 16 |  | 28 | 38 | 33.5 | 11.2 |  |
|  | 35-50 | 10YR 2/1 | 4.46 |  | -- | 101 | 213 | -- |  | 2.28 |  | 61.3 |  | 11 | 0.7 | 17 |  | 47 | 6.8 | 46.2 | 2.97 |  |
|  | 50-65 | 2.5Y 3/3 | 4.30 |  | 673 | 399 | -- | -- |  | 1.67 |  | 77.8 |  | 19 | 0.9 | 21 |  | 17 | 42 | 51.1 | 17.6 |  |
|  | 65-80 | 10YR 2/1 | 4.06 |  | -- | 669 | 618 | -- |  | 2.81 |  | 48.7 |  | 14 | 0.7 | 19 |  | 12 | 52 | 36.1 | 4.72 |  |
| LCP  Center | 0-21 | 10YR 2/1 | 4.68 |  | -- | -- | 365 | 341 |  | 10.6 |  | 91.2 |  | 39 | 1.8 | 23 |  | -- | -- | -- | -- |  |
|  | 21-49 | 2.5YR 2.5/1 | 5.19 |  | -- | 113 | 86.5 | 87.7 |  | 1.17 |  | 54.2 |  | 16 | 0.7 | 22 |  | 34 | 14 | 52 | 0.57 |  |
|  | 49-67 | 10YR 2/1 | 5.36 |  | -- | 104 | 71.5 | 79.9 |  | -- |  |  |  | 17 | 0.8 | 21 |  | 33 | 14 | 53.4 | 4.94 |  |
| ^1^Measured gravimetric water content (g H_2_O g^-1^ dry weight soil); ^2^Modeled volumetric water content by HYPROP (cm^3^ H_2_O cm^-3^ soil); ^3^Soil texture 100% = clay + silt + sand and >2mm (mostly plant debris) reported as wt.% of total sample; *ratios are for different soil:DI water ratios. | | | | | | | | | | | | | | | | | | | | | | |

| **Table S3 \|** Soil water properties of HCP and LCP cores determined by fitting the unimodal van Genuchten-Mualem model to integrated HYPROP and WP4 measurements. | | | | | | | | | | | | | | | |
| --- | --- | --- | --- | --- | --- | --- | --- | --- | --- | --- | --- | --- | --- | --- | --- |
| Micro- Topography | Depth | Initial θ_s_ | Bulk density  (g/cm^3^) | Porosity |  | Retention Function Parameters | | | |  | Conductivity Function Parameters | |  | Statistical Analysis |  |
|  |  |  |  |  |  |  |  |  |  |  |  |  |  |  |  |
|  | (cm) | (%) |  |  |  | *α* | *n* | *θ_r_* | *θ_s_* |  | *K_s_* | *τ* |  | AICc |  |
|  |  |  |  |  |  | (1/cm) |  | (cm^3^/cm^3^) | (cm^3^/cm^3^) |  | (cm/d) |  |  |  |  |
| HCP  Center | 0-10 | 64.9 | 0.71 | 0.73 |  | 0.09 | 1.2 | 0.003 | 0.605 |  | 38.2 | -5.833 |  | -855 |  |
|  | 10-20 | 65.5 | 0.53 | 0.8 |  | 0.05 | 1.24 | 0 | 0.654 |  | 10.7 | -1.066 |  | -1363 |  |
|  | 20-35 | 64.4 | 0.75 | 0.72 |  | 0.018 | 1.33 | 0.021 | 0.649 |  | 54.3 | -0.318 |  | -1176 |  |
|  | 35-50 | 61.3 | 0.8 | 0.7 |  | 0.029 | 1.27 | 0 | 0.601 |  | 1309 | 1.364 |  | -1202 |  |
|  | 50-65 | 77.8 | 0.44 | 0.83 |  | 0.06 | 1.41 | 0.018 | 0.81 |  | 10000 | 3.376 |  | -1331 |  |
|  | 65-80 | 48.7 | 0.18 | 0.93 |  | 0.381 | 1.3 | 0.031 | 0.737 |  | 10000 | 1.163 |  | -1180 |  |
| LCP  Center | 0-21 | 91.2 | 0.09 | 0.97 |  | 0.15 | 1.38 | 0.013 | 1 |  | 1806.5 | 1.943 |  | -1222 |  |
|  | 21-49 | 54.2 | 1.02 | 0.62 |  | 0.004 | 1.34 | 0.035 | 0.516 |  | 16.1 | 1.595 |  | -1330 |  |
|  | 49-67 | >99 |  |  |  |  |  |  |  |  |  |  |  |  |  |
| Water retention measurements were fit to the unimodal model of van Genuchten (1980) with shape parameters α and n, residual water content θ_r_, and saturated water content θ_s_. The saturated hydraulic conductivity K_s_, and tortuosity parameter τ, were fit concurrently. AICc, Akaike Information Criterion. | | | | | | | | | | | | | | | |

| **Table S4 \|** Response function fitting parameters for CO_2_ production from HCP microcosms. Calculated values and statistics of the fit parameters (A, B and d) obtained when $\boldsymbol{C}$ (µmol CO_2_ g^-1^) values were fitted to hyperbolic: $\boldsymbol{C=A}\frac{\boldsymbol{t}}{\boldsymbol{(B+t)}}$, sigmoidal:$\boldsymbol{C= A}\frac{\boldsymbol{t}^{\boldsymbol{d}}}{{\boldsymbol{B}^{\boldsymbol{d}}\boldsymbol{+t}}^{\boldsymbol{d}}}$, or exponential:$\boldsymbol{C=A}\boldsymbol{e}^{\boldsymbol{Bt}}$ . Values from LCP are reported elsewhere (Roy Chowdhury et al., 2015). | | | | | | | | | | |
| --- | --- | --- | --- | --- | --- | --- | --- | --- | --- | --- |
| Micro-topography | Soil Horizon | Temperature (^o^C) | Model | A | 95% Confidence Interval | B | 95% Confidence Interval | d | 95% Confidence Interval | R^2^ |
| HCP  Center | Organic | 8 | Exponential | 3.811 | 2.925-4.782 | 0.03914 | 0.03424-0.04458 | -- | -- | 0.910 |
|  |  | 4 | Exponential | 1.859 | 1.491-2.261 | 0.04793 | 0.04390-0.05236 | -- | -- | 0.967 |
|  |  | -2 | Exponential | 0.8923 | 0.7280-1.065 | 0.02271 | 0.01819-0.02748 | -- | -- | 0.763 |
|  | Mineral | 8 | Exponential | 0.7220 | 0.4896-0.9917 | 0.05916 | 0.05301-0.06653 | -- | -- | 0.969 |
|  |  | 4 | Hyperbolic | 6.658 | 4.331-16.26 | 101.1 | 53.33-305.2 | -- | -- | 0.958 |
|  |  | -2 | Hyperbolic | 1.997 | 1.606-2.670 | 38.29 | 25.25-62.35 | -- | -- | 0.950 |
| HCP  Trough | Organic | 8 | Sigmoidal | 46.07 | 41.88-53.45 | 5.691 | 4.348-7.901 | 1.665 | 1.078-2.582 | 0.9323 |
|  |  | 4 | Sigmoidal | 38.01 | 35.20-47.67 | 8.034 | 7.032-11.46 | 4.677 | 1.500-9.118 | 0.9301 |
|  |  | -2 | Sigmoidal | 28.55 | 23.29-51.92 | 15.89 | 11.38-50.10 | 1.596 | 0.9006-2.547 | 0.9294 |
|  | Mineral | 8 | Hyperbolic | 3.38 | 2.516-5.219 | 24.14 | 11.58-55.04 | -- | -- | 0.831 |
|  |  | 4 | Exponential | 0.3054 | 0.1845-0.4501 | 0.05017 | 0.04164-0.06073 | -- | -- | 0.936 |
|  |  | -2 | Hyperbolic | 0.9186 | 0.6752-1.488 | 7.129 | 1.286-28.45 | -- | -- | 0.968 |

| **Table S5 \|** Response function fitting parameters for CH_4_ production from HCP trough microcosms. CH_4_ production was not observed from HCP center soils. Calculated values and statistics of the fitted parameters (A, B and d) obtained when $\boldsymbol{C'}$ (µmol CH_4_ g^-1^) values were fit to the sigmoidal model:$\boldsymbol{C'= A}\frac{\boldsymbol{t}^{\boldsymbol{d}}}{{\boldsymbol{B}^{\boldsymbol{d}}\boldsymbol{+t}}^{\boldsymbol{d}}}$. No CH_4_ was detected in the Center of HCP. Values from LCP are reported elsewhere (Roy Chowdhury et al., 2015). | | | | | | | | | | |
| --- | --- | --- | --- | --- | --- | --- | --- | --- | --- | --- |
| Micro-topography | Soil Horizon | Temperature (^o^C) | Model | A | 95% Confidence Interval | B | 95% Confidence Interval | d | 95% Confidence Interval | R^2^ |
| HCP  Trough | Organic | 8 | Sigmoidal | 2.369 | 2.121-2.748 | 16.89 | 14.62-19.73 | 4.396 | 2.455-11.19 | 0.9250 |
|  |  | 4 | Sigmoidal | 1.854 | 1.720-2.071 | 18.39 | 17.22- 19.85 | 7.357 | 3.322-12.73 | 0.9590 |
|  |  | -2 | Sigmoidal | 0.03813 | -- | 7.030 | -- | 78.57 | -- | 0.4776 |
|  | Mineral | 8 | Sigmoidal | 5.536 | 3.993-9.149 | 25.32 | 19.94-54.24 | 2.280 | 1.435-3.521 | 0.9210 |
|  |  | 4 | Sigmoidal | 3.246 | 2.354-24.38 | 30.40 | 21.50-317.2 | 2.105 | 1.156-3.580 | 0.9340 |
|  |  | -2 | Sigmoidal | 0.944 | 0.8155-1.378 | 16.2 | 13.28-28.72 | 2.261 | 1.244-3.805 | 0.9340 |

## Supplementary Figures

**Figure S1 |** High-centered polygon (HCP) **(A)** has a raised center surrounded by a trough that is usually saturated with water; low-centered polygon (LCP) **(B)** has an intermittently saturated center that is surrounded by an elevated rim (or ridge) and a low-lying trough that is usually saturated with water. Gravimetric water content of center **(C)** and trough **(D)** soils used in microcosm experiments were measured at fixed depth intervals, plotted in units of g H_2_O g^-1^ dwt. soil. Moisture content of HCP center **(E)** measured in the field with a 5TE sensor attached to a ProCheck meter (Decagon Devices). LCP data win C and D from previous study with core (NGADG0013) from N 71° 16.8932′, W 156° 36.6200 (Roy Chowdhury et al., 2015).


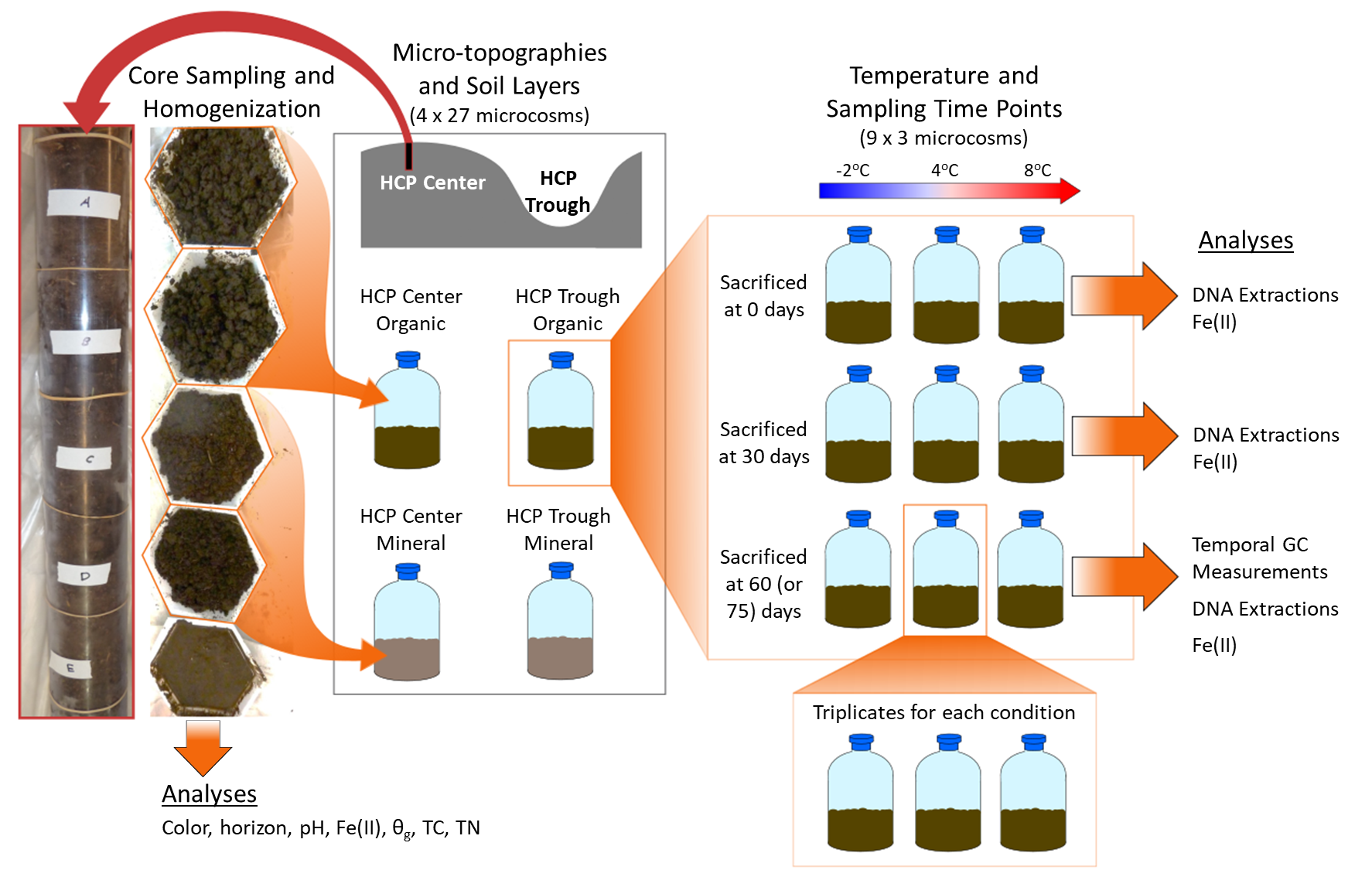


**Figure S2 |** Soil microcosm incubations for HCP soils. Photos of HCP center core homogenization shown on left with initial analyses denoted. HCP trough organic microcosms are shown in detail as an example of microcosm conditions for each micro-topography + soil layer condition. Microcosms were sacrificed at 0, 30, and 60 or 75 days for analyses. All conditions were evaluated in triplicate.

**
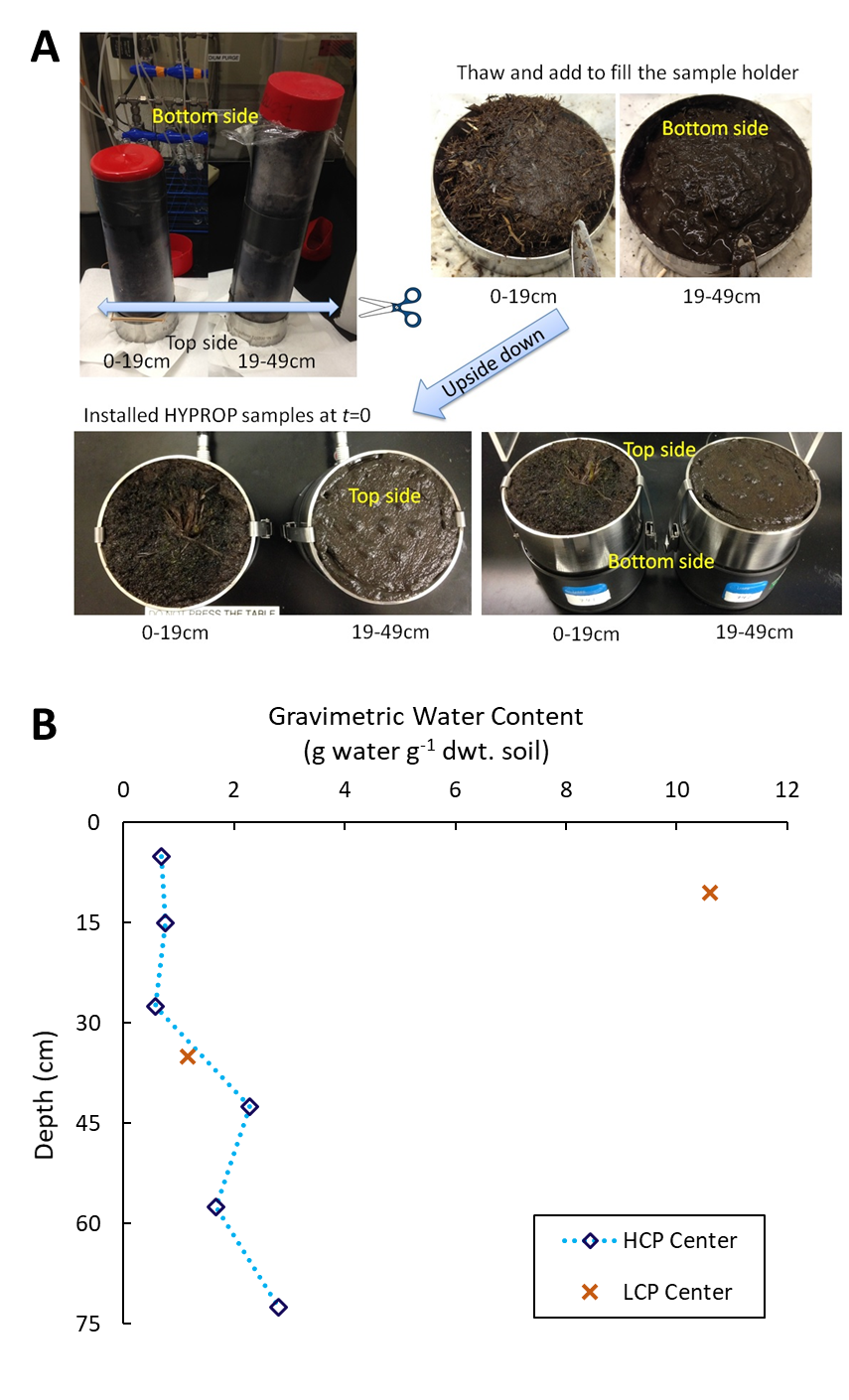
**

**Figure S3 |** Sample preparation of core sections **(A)** for water potential measurements with HYPROP sample ring set up (images from LCP center samples), and gravimetric water content measurements **(B)** for the HCP and LCP center cores used for water potential measurements. LCP data presented previously (Roy Chowdhury et al., 2015).


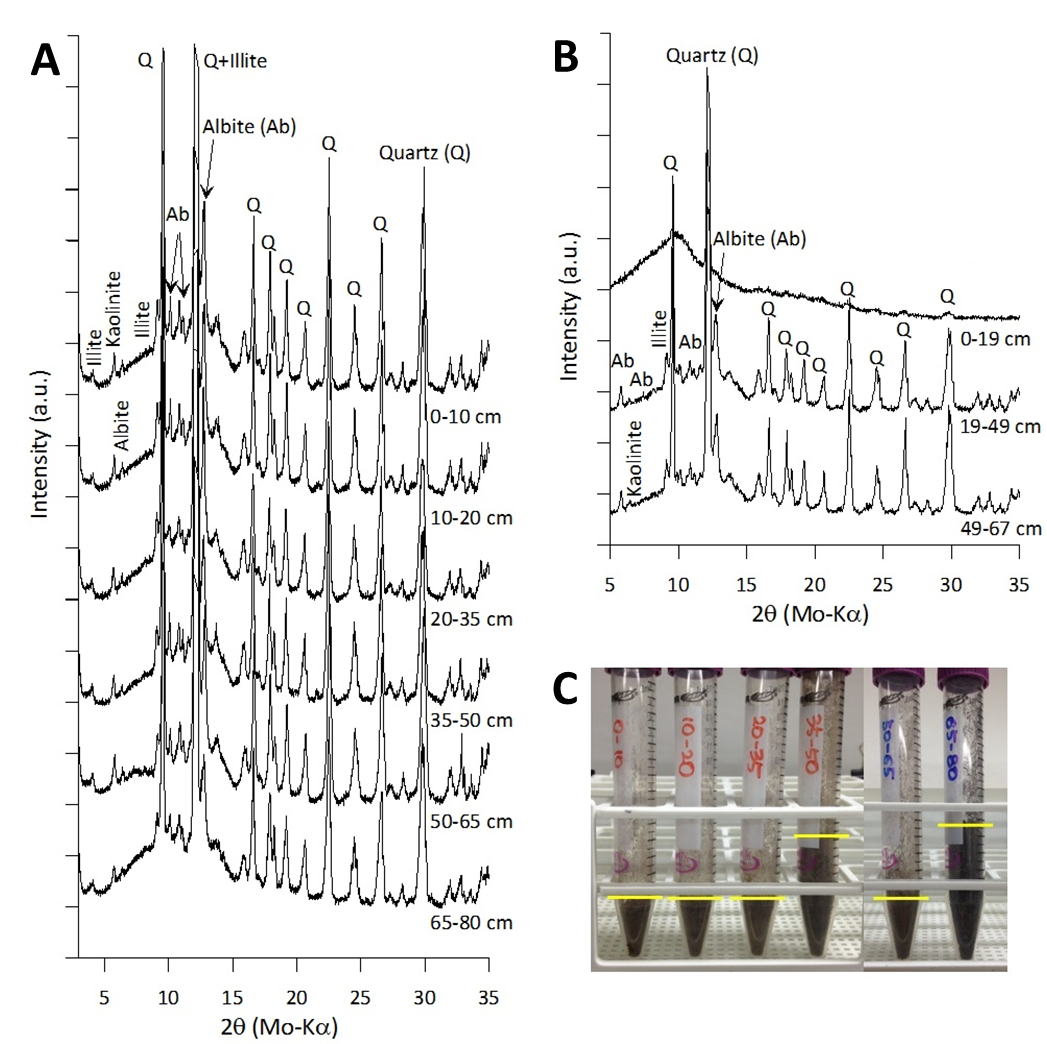


**Figure S4 |** XRD results from the core sections of HCP center **(A)** and LCP center **(B)** soils. Picture of HCP samples **(C)** showing differences in soil volumes for samples of the same dry weight equivalent.

**
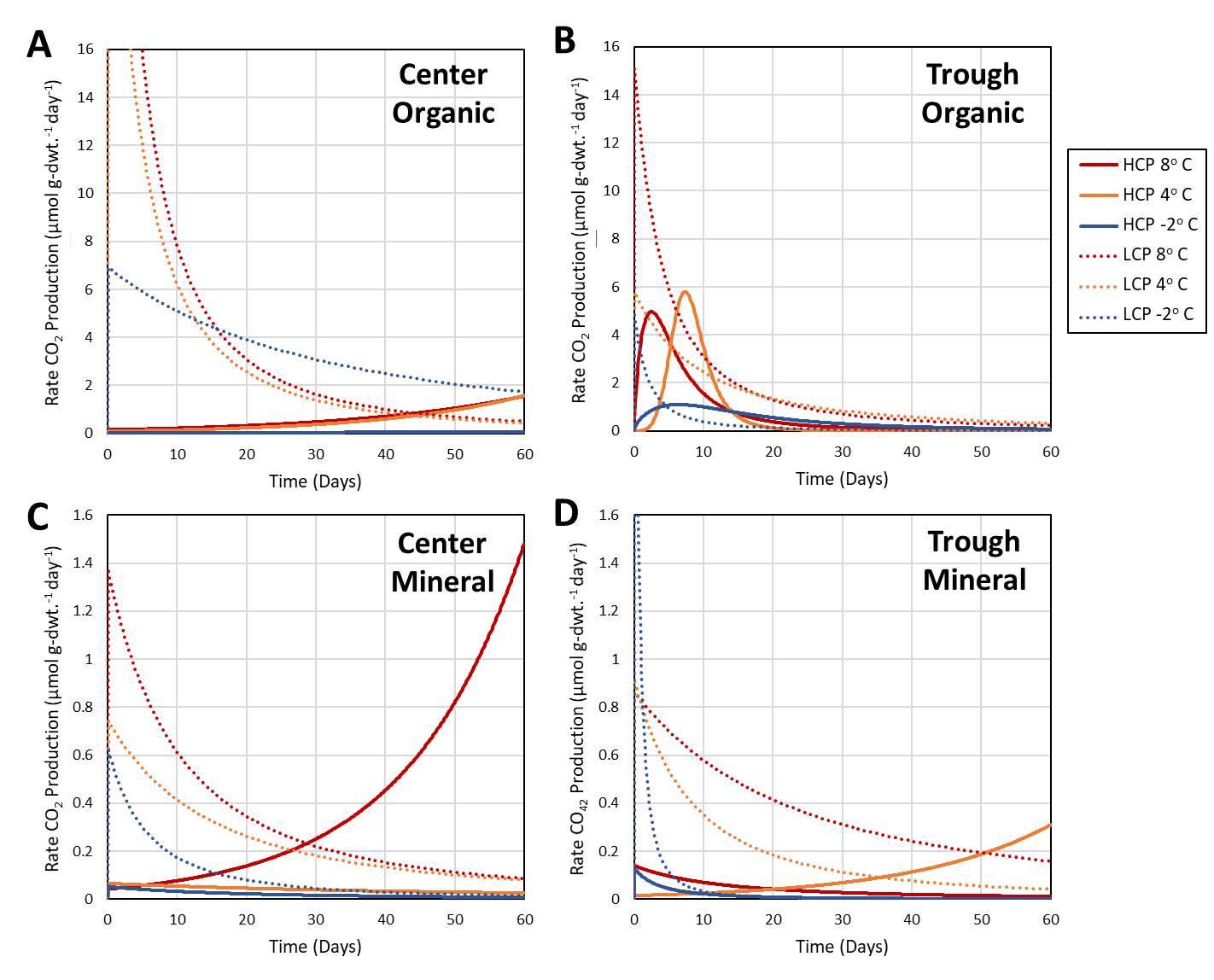
**

**Figure S5 |** Calculated rates of CO_2_ production in polygon soils from organic center **(A)** and trough **(B)**, as well as mineral center **(C)** and trough **(D)**. Solid lines represent the derivative of CO_2_ response curves for HCP soils, and dashed lines represent the derivative of CO_2_ response curves for LCP soils. Parameters for HCP response curves are presented in Table S4 and LCP parameters were presented previously (Roy Chowdhury et al., 2015).


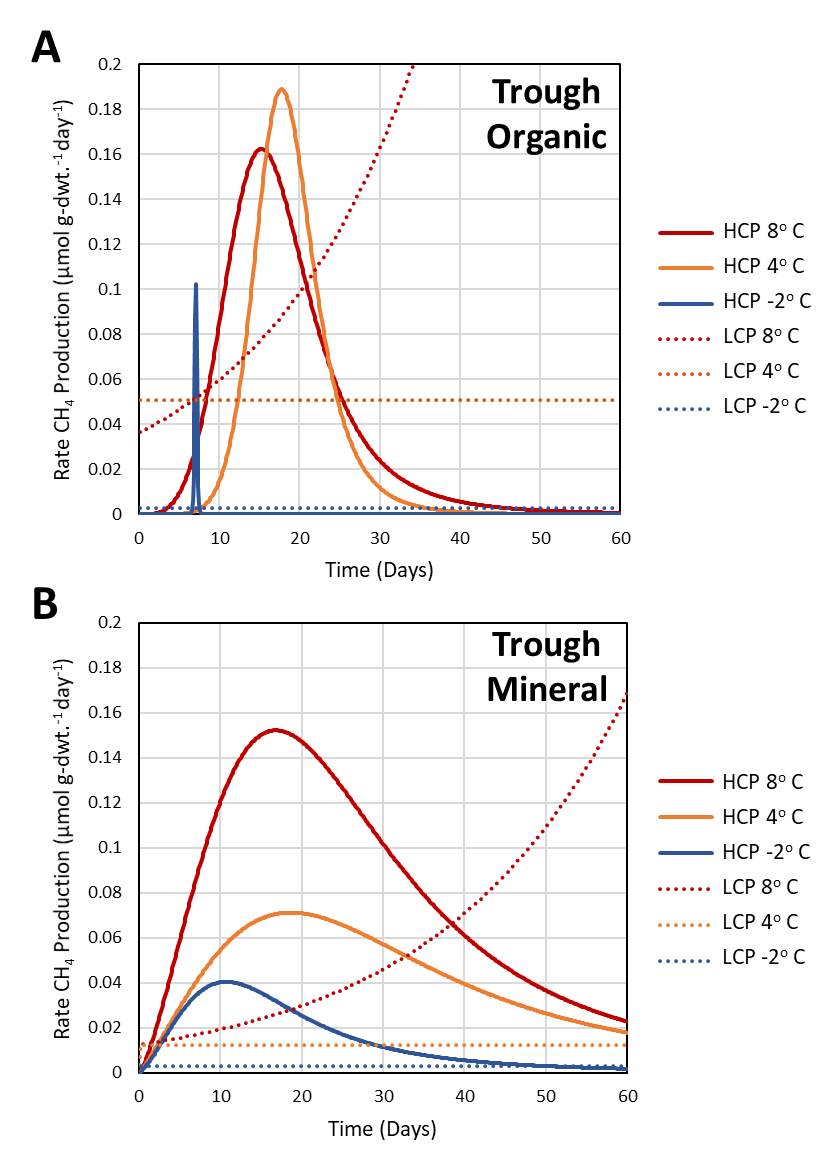


**Figure S6 |** Rates of CH_4_ production in polygon soils from organic **(A)** and mineral **(B)** soils of HCP (solid lines) and LCP (dashed lines) troughs. Rates are derivatives of the response curve functions for CH_4_ production. Parameters for HCP response curves are presented in Table S5 and LCP parameters were presented previously (Roy Chowdhury et al., 2015). No CH_4_ was detected in HCP center samples.


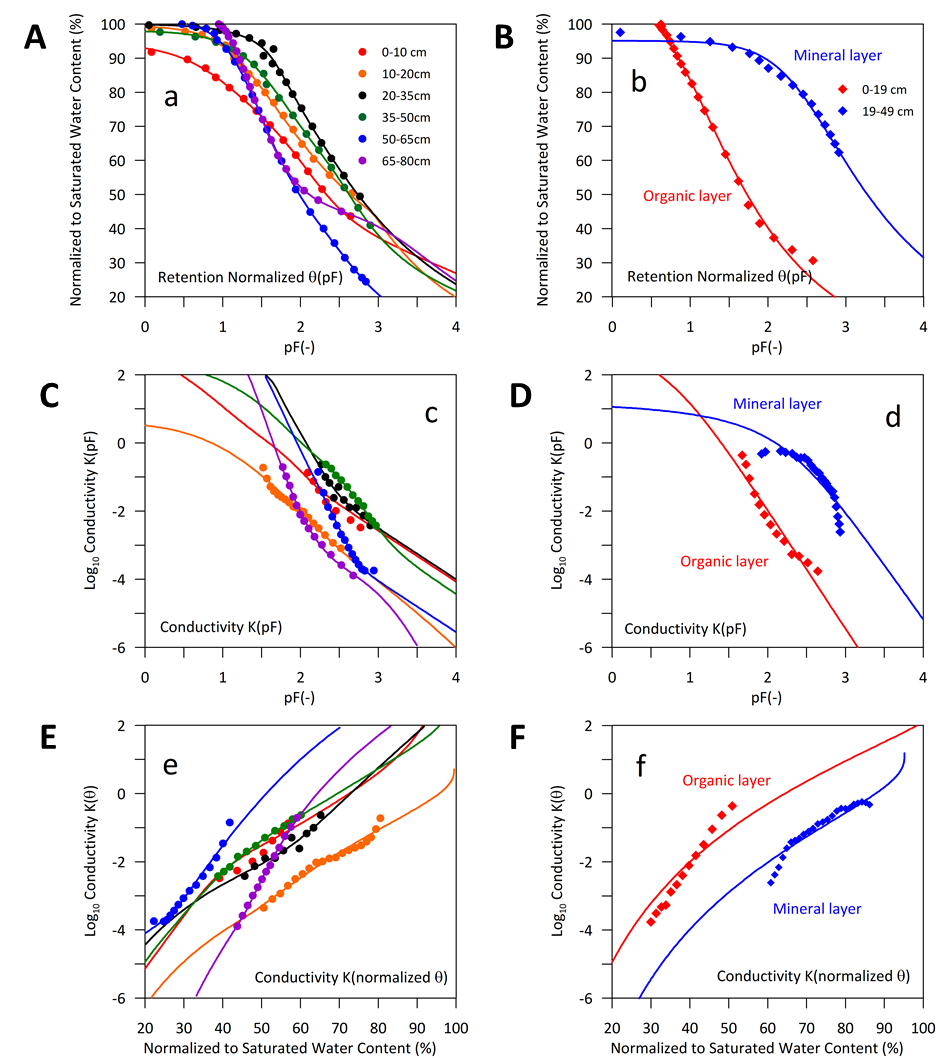


**Figure S7 |** Correlation between normalized saturated water content (θs), hydraulic conductivity (K), and soil water pressure (pF), where pF = log(-h) [h is head in cm], fitted using the van Genuchten model in the HCP center **(A, C, & E)** and LCP center cores **(B, D, & F)**. LCP center (NGADG0013) was sampled at N 71° 16.8932′, W 156° 36.6200′. Note: water contents are normalized to saturated water content, and axes for A and B are flipped from orientation in Figure 3 of manuscript.


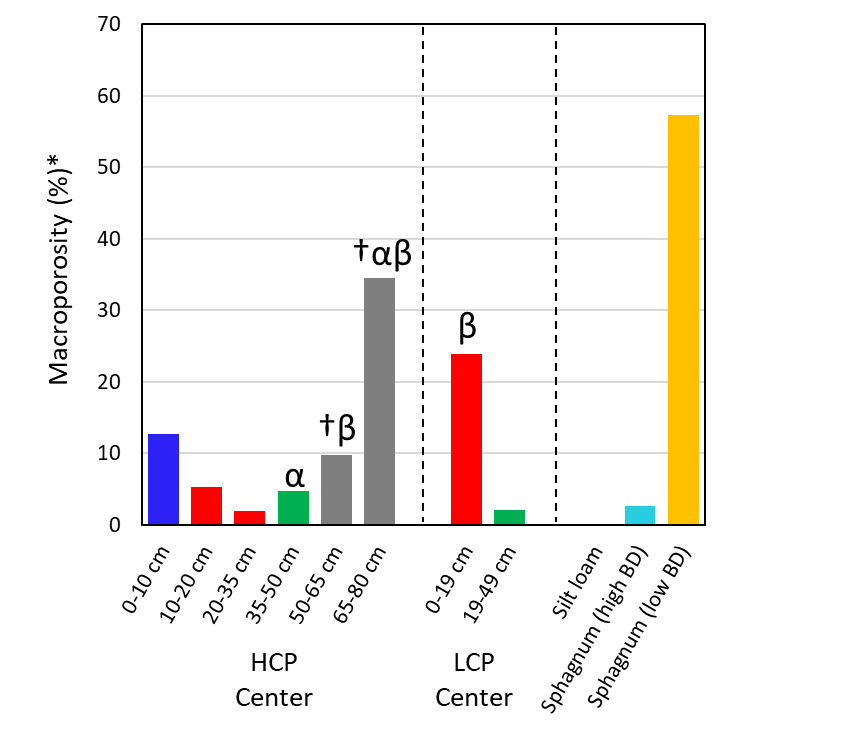


**Figure S8 |** Macropore distribution for HCP and LCP center soils determined from soil water retention curves.

* = percent pore volume extracted (beginning with saturation) under 10^1^ hPa matric potential

† = high volume of ice; macropore estimates likely include voids from ice lenses

α = interbedded organic layers present

β = utilized model fit θ_s_ for macroporosity calculation (opposed to measured maximum θ_v_)


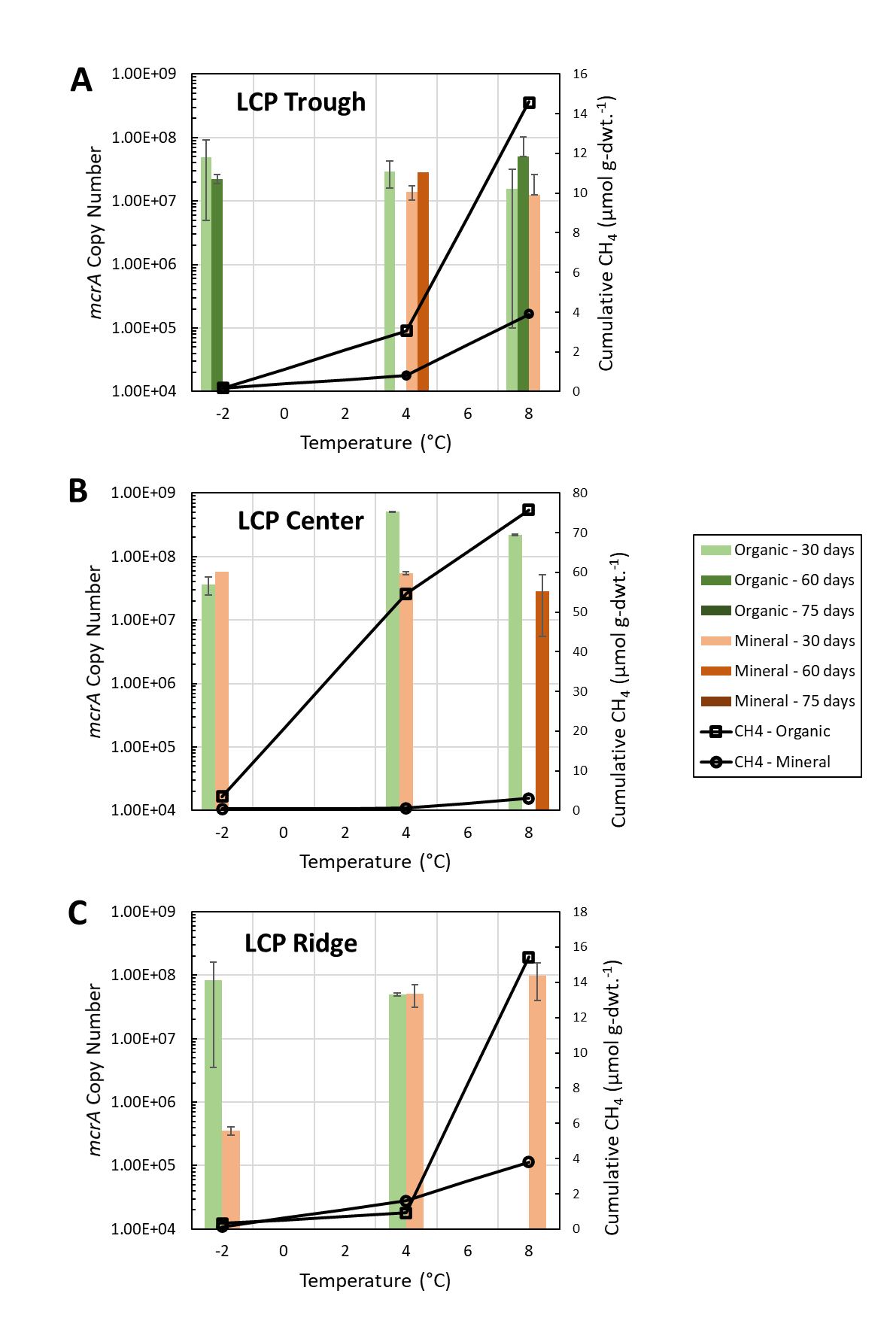


**Figure S9 |** *mcrA* copy numbers for LCP organic and mineral soils determined from DNA extracted at either 30, 60, or 75 days of incubation. Copy numbers are plotted on a log scale (primary vertical axis). Temperatures on the horizontal axis are plotted on a linear scale (as are the cumulative CH_4_ values plotted on the secondary vertical axis).

Brooks, R.H., and Corey, A.T. (1964). Hydraulic properties of porous media. *Hydrology papers (Colorado State University); no. 3*.

Fredlund, D.G., and Xing, A. (1994). Equations for the soil-water characteristic curve. *Canadian geotechnical journal* 31**,** 521-532.

Kosugi, K.I. (1996). Lognormal distribution model for unsaturated soil hydraulic properties. *Water Resources Research* 32**,** 2697-2703.

Roy Chowdhury, T., Herndon, E.M., Phelps, T.J., Elias, D.A., Gu, B., Liang, L., Wullschleger, S.D., and Graham, D.E. (2015). Stoichiometry and temperature sensitivity of methanogenesis and CO2 production from saturated polygonal tundra in Barrow, Alaska. *Global Change Biology* 21**,** 722-737.

Van Genuchten, M.T. (1980). A closed‐form equation for predicting the hydraulic conductivity of unsaturated soils. *Soil Science Society of America Journal* 44**,** 892-898.

## Supplementary Material References
